# Source‐to‐Sink Terrestrial Analogs for the Paleoenvironment of Gale Crater, Mars

**DOI:** 10.1029/2020JE006530

**Published:** 2021-02-18

**Authors:** Michael T. Thorpe, Joel A. Hurowitz, Kirsten L. Siebach

**Affiliations:** ^1^ Department of Earth, Environmental and Planetary Sciences Rice University Houston TX USA; ^2^ NASA Johnson Space Center NASA Postdoctoral Program Houston TX USA; ^3^ Department of Geosciences State University of New York at Stony Brook Stony Brook NY USA

**Keywords:** basaltic weathering, Mars paleoclimate, terrestrial analogs

## Abstract

In the Late Noachian to Early Hesperian period, rivers transported detritus from igneous source terrains to a downstream lake within Gale crater, creating a stratified stack of fluviolacustrine rocks that is currently exposed along the slopes of Mount Sharp. Controversy exists regarding the paleoclimate that supported overland flow of liquid water at Gale crater, in large part because little is known about how chemical and mineralogical paleoclimate indicators from mafic‐rock dominated source‐to‐sink systems are translated into the rock record. Here, we compile data from basaltic terrains with varying climates on Earth in order to provide a reference frame for the conditions that may have prevailed during the formation of the sedimentary strata in Gale crater, particularly focusing on the Sheepbed and Pahrump Hills members. We calculate the chemical index of alteration for weathering profiles and fluvial sediments to better constrain the relationship between climate and chemical weathering in mafic terrains, a method that best estimates the cooler limit of climate conditions averaged over time. We also compare X‐ray diffraction patterns and mineral abundances from fluvial sediments in varying terrestrial climates and martian mudstones to better understand the influence of climate on secondary mineral assemblages in basaltic terrains. We show that the geochemistry and mineralogy of most of the fine‐grained sedimentary rocks in Gale crater display first‐order similarities with sediments generated in climates that resemble those of present‐day Iceland, while other parts of the stratigraphy indicate even colder baseline climate conditions. None of the lithologies examined at Gale crater resemble fluvial sediments or weathering profiles from warm (temperate to tropical) terrestrial climates.

## Key Points

- Terrestrial sediments and weathering profiles from basaltic terrains display first‐order similarities with mudstones from Gale crater, Mars
- The chemical index of alteration can be used as paleoclimate proxy and places constraints on the ancient climate of Gale crater, Mars
- The paleoclimate of Gale crater, Mars, was variable and ranged from Icelandic‐like conditions to colder climates

## 1 Introduction

Preserved in the sedimentary rock record of Mars is an ancient (>3 Ga) history of fluvial activity that persisted long enough to create landforms and layered sequences that suggest both subaerial and subaqueous deposition (Malin & Edgett, 2000, 2003). Geomorphological features observed from orbit provide evidence for extensive fluviolacustrine processes, as evidenced by channelized flow features and delta‐like distributary fans (Hynek et al., 2010; Milton, 1973; Sharp & Malin, 1975). On the ground, sedimentological observations by the *Curiosity* rover include conglomerates, sandstones, and mudstones interpreted to have fluviolacustrine origins (e.g., Grotzinger et al., 2014, 2015). Geochemical and mineralogical investigations of these fluviolacustrine sedimentary rocks have revealed sediment interaction with surface and ground waters in a dynamic weathering environment (e.g., Bristow et al., 2015; Hurowitz et al., 2017; Mangold et al., 2019; McLennan et al., 2014, 2019; Rampe, Ming, et al., 2017; Rampe et al., 2020; Vaniman et al., 2014). These deposits, which date from a period of time that has been largely erased from the Earth's sedimentary record by plate tectonics and intracrustal melting (McLennan & Grotzinger, 2008; Veizer & Jansen, 1985), have the potential to provide insight into the early geological history of our Solar System (McLennan et al., 2019). However, without a detailed understanding of the climate that Gale crater sedimentary strata were deposited under, and importantly, the ways that climate would have acted to modify sediment composition during transport and deposition, significant uncertainty can be introduced when those compositions are used to infer and constrain regional, intraplanetary and interplanetary processes.

Chemical weathering is perhaps the most significant process influencing the composition of sediments and sedimentary rocks on both Earth and Mars. There have been numerous attempts to use bulk chemical composition to quantify the degree of chemical weathering in sedimentary rocks (e.g., Babechuk et al., 2014; Fedo et al., 1995; Harnois, 1988), but the chemical index of alteration (CIA; H. Nesbitt & Young, 1982) remains the most widely employed index in studies concerned with reconstructing paleoweathering conditions from bulk geochemistry. The CIA is a molar ratio that juxtaposes the behavior of an immobile element (Al, expressed as the oxide Al_2_O_3_) against those elements easily mobilized during incongruent weathering of minerals and glass by aqueous solutions (Equation 1).

(1) $$\mathrm{CIA}=100\times \frac{{\mathrm{Al}}_{2}O_{3}}{{\mathrm{Al}}_{2}O_{3}+\mathrm{CaO}+{\mathrm{Na}}_{2}O+K_{2}O}$$

When applied to sedimentary rocks, the CIA (like other similar weathering indices) is thought to reflect the integrated effects of chemical weathering experienced by the mineral constituents in those rocks during erosion and transport, and after deposition. The extent of chemical weathering is largely controlled by climatic variables, that is, temperature, amount of precipitation, and form of precipitation (e.g., P. Dinis et al., 2017; P. A. Dinis et al., 2019; Guo et al., 2018; H. W. Nesbitt, 2003), and thus the CIA has been successfully employed as a paleoclimate proxy for sedimentary rocks, even in terrestrial eons when atmosphere and surface conditions were quite different than the present. For example, H. Nesbitt and Young (1982) used CIA values from Huronian sedimentary rocks to unravel climate variations ranging from tropical to glacial during the Proterozoic (2.1–2.5 Gya). The CIA has also been used to assess the degree of weathering on a catchment scale to evaluate the influence of the contemporary climate on chemical weathering. For example, in a survey of suspended sediment composition in catchments from around the globe, C. Li and Yang (2010) investigated the sensitivity of CIA to climate variables and concluded that CIA is a reliable proxy for evaluating the integrated weathering history of drainage basins. These examples are two of many that highlight how climate and chemical weathering are closely tied and are translated into the sedimentary rock record via the CIA, ultimately providing confidence that this proxy can be used to understand the paleoclimate of ancient Mars from the compositions of sedimentary rocks analyzed by current and future in situ instruments.

Despite significant evidence for chemical weathering on the martian surface (e.g., Ehlmann & Edwards, 2014; McLennan et al., 2019), a complication is introduced by differences in the average provenance composition of sediments and sedimentary rocks between Earth and Mars. Regardless of provenance, G. Li et al. (2016) showed that chemical weathering rates are correlated with climate all around the Earth. However, in more regional or small‐scale assessments, provenance becomes increasingly important. On Earth, the exposed crust is dominated by geochemically evolved granodiorites and the sediments derived from them (Taylor & McLennan, 1985), whereas Mars' exposed crust is basaltic (Taylor & McLennan, 2009). Fewer studies of sediments and sedimentary rocks have been conducted in basaltic terrains on Earth because subaerial exposures of basalts are only a minor fraction of the crust available for chemical weathering. Furthermore, changes in CIA during incipient alteration of a basaltic progenitor are understudied on Earth and this, in turn, complicates the interpretation of alteration history in the sedimentary rocks in Gale crater. For example, the *Curiosity* rover investigated the Sheepbed formation early in its traverse and discovered that these lacustrine mudstones contain significant amounts of juvenile weathering products (i.e., smectitic clays and amorphous phases, Rampe, Ming, et al., 2017; Vaniman et al., 2014) but their CIA values have led to multiple interpretations, all of which have paleoclimate implications. One scenario envisioned a frigid climate resulting in little to no chemical weathering prior to deposition, and mineralogical transformations occurring during diagenesis in a closed system (Bristow et al., 2015; McLennan et al., 2014). In contrast, a second scenario proposed weathering in the source terrains and a detrital origin for clay minerals, assuming salts added labile cations back into the system after deposition, which would require comparatively more clement conditions (Schieber et al., 2017). Perhaps, the more surprising finding is that these juvenile weathering products, commonly observed in modern fluvial sediments on Earth (e.g., M. T. Thorpe et al., 2019), survived weathering, transport, and diagenesis in sedimentary rocks that are 3.5 billion years old on the surface of Mars, whereas similar products typically only survive for millions of years on Earth (Tosca & Knoll, 2009). These discrepancies and knowledge gaps demonstrate where terrestrial analog studies can shed light on the sedimentary history of Mars.

The goal of this contribution is to provide improved constraints on the paleoclimate of Gale crater, specifically focusing on the paleoclimate present during the sedimentation of the Sheepbed and Pahrump Hills members of the Bradbury and Mount Sharp groups (Grotzinger et al., 2014, 2015), respectively. We have compiled relevant geochemical records derived from different terrestrial climates to facilitate a comparison between the chemistry of sedimentary rocks from Gale crater, Mars, and basaltic sediments on Earth. We place a particular emphasis on comparisons to river systems situated in the Columbia River Basalt (CRB) group (Idaho, USA) and in Iceland, which have catchments that are dominated by a basaltic provenance, and fluvial systems that contain deposits of first‐cycle basaltic sediments, and for which we also have detailed mineralogical observations (M. T. Thorpe & Hurowitz, 2020; M. T. Thorpe et al., 2019). These terrestrial watershed attributes make them reasonable potential analogs for an ancient fluvial source‐to‐sink system in Gale crater. Furthermore, the climates of Iceland and Idaho span a range of proposed martian climate scenarios, providing a means to compare and contrast the geochemistry and mineralogy of sediments generated in the (i) relatively cold and icy conditions of Iceland against the (ii) comparatively warmer and wetter environment of northwest Idaho, USA. Accordingly, this work will address the geochemical and mineralogical transformations that result from source‐to‐sink processes in basaltic watersheds and ultimately provide a useful reference frame from which to interpret the sedimentary paleoclimate records preserved in Gale crater, and eventually Jezero crater, which is the landing site for the upcoming Mars 2020 *Perseverance* Rover mission.

## 2 Data Sources

**Figure 1:**
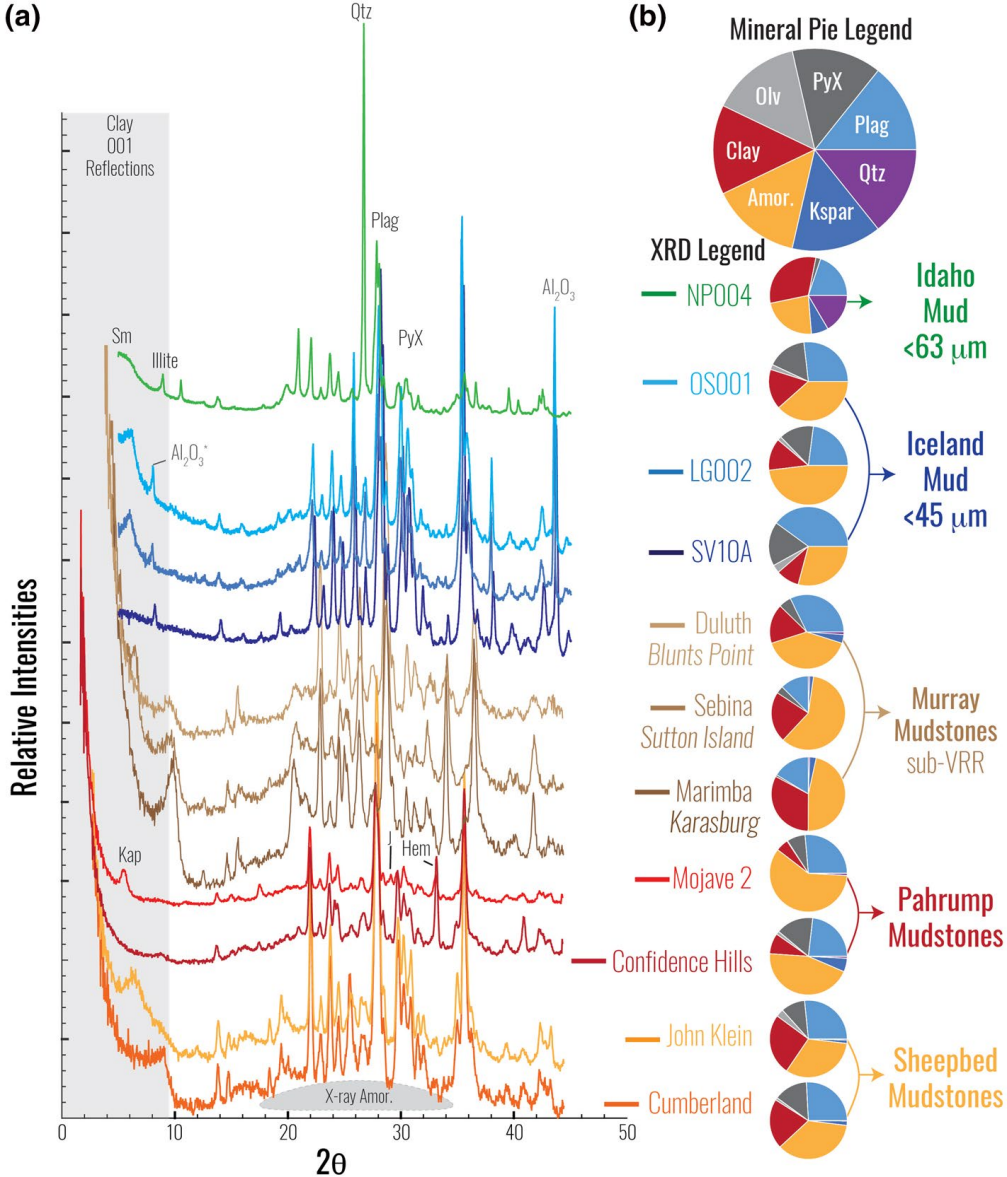
XRD patterns for two Sheepbed mudstones (Vaniman et al., 2014), two Pahrump Hills mudstones (Rampe, Ming, et al., 2017), three mudstones from the Murray formation between Pahrump Hills and Vera Rubin ridge (sub‐VRR) (Bristow et al., 2018; Rampe et al., 2020), the <45‐μm sediment from Iceland (M. T. Thorpe et al., 2019), and a <63‐μm sediment sample from the Idaho (M. T. Thorpe & Hurowitz, 2020) sample suite (a). Martian target names and terrestrial analog sample IDs are next to the XRD patterns. In (b), pie charts display the mineral group abundances for these terrestrial muds and martian mudstones. First‐order similarities are observed in the XRD patterns for Iceland and Gale crater mudstones, including clay mineral reflections at low‐two theta, sharp, well‐resolved peaks from primary minerals (esp., plagioclase and pyroxene) sharp peaks, and an elevated background from the scatter produced from an X‐ray amorphous phase(s). Mineral abundances between Iceland mud and Gale crater mudstones also show similarities. XRD pattern and pie chat abbreviations: plagioclases (Plag); pyroxenes (Pyx); olivines (Olv); smectites (sm); smectite, illite, and kaolinite (Clay); quartz (Qtz); X‐ray amorphous (X‐ray Amor or just Amor); hematite (Hem); jarosite (J); kapton window (Kap); Al_2_O_3_ internal standard in Icelandic samples accounts for <1 wt% of total derived mineral abundances.

Since landing in Gale crater, *Curiosity* has traversed over ∼400 m of a thick sedimentary sequence, investigating and sampling lithified fluvial and lacustrine deposits from the Bradbury and Mount Sharp groups (Figure S1). The stratigraphy of the Bradbury group is predominantly fluvial in origin, except for a few finer‐grained intervals (e.g., Sheepbed member of the Yellowknife Bay formation, *see also* Figure S2). The Mount Sharp group, on the other hand, is dominated by lacustrine mudstone (Fedo et al., 2019; Grotzinger et al., 2014, 2015; Stack et al., 2019; Edgar et al., 2020). The major element geochemistry of martian rocks was obtained using the Alpha‐Particle X‐ray Spectrometer (APXS) onboard the *Curiosity* rover; the subset of targets discussed in this study was compiled from published sources, including: (i) 41 mudstone analyses from the Sheepbed member of the Yellowknife Bay (YKB) formation (McLennan et al., 2014) and (ii) 44 analyses of mudstones from the hematite–phyllosilicate (HP) facies at the Pahrump Hills member of the Murray Formation (Hurowitz et al., 2017). In addition to these sedimentary rock samples, an estimate for the elemental composition of the martian crust, derived from the soil chemistry of landed missions and orbital chemistry from gamma ray spectroscopy, was also included and used as a general proxy for the provenance of Gale crater sedimentary rocks (Hahn & McLennan, 2007; Taylor & McLennan, 2009). Mineral abundances were modeled based on powder diffraction patterns acquired by the X‐ray diffractometer onboard *Curiosity*, the Chemistry and Mineralogy (CheMin) instrument, with data compiled for (i) two mudstone analyses from the Sheepbed member from the Bradbury group (Vaniman et al., 2014) and (ii) two mudstone analyses from the Murray HP facies from the Mount Sharp group (Rampe, Ming, et al., 2017). All martian data compiled for this contribution are available on the Planetary Data System Geoscience Node (http://pds‐geosciences.wustl.edu/missions/msl/index.htm; see also Gellert et al., 2015) and tabulated and further explained in Tables S1 and S2. While the Sheepbed and Pahrump Hills members are the focus of this work, more recent *Curiosity* targets in the Murray formation are also included to demonstrate the utility in extending this terrestrial reference frame. However, the nature of the sedimentary rocks higher in the strata of the Murray formation is complicated by more significant diagenetic overprints (e.g., Fraeman et al., 2020; Rampe et al., 2020) as well as diverse amorphous compositions (e.g., Achilles et al., 2020), both of which obscure geochemical signatures from subaerial weathering (Siebach & McLennan, 2018). Therefore, we only include the APXS geochemistry for the target with the highest uncorrected CIA value from the Karasburg, Sutton Island, and Blunts Point members (Berger et al., 2020), which are less impacted by these complicating factors, as well as the APXS analyses (Berger et al., 2020) and CheMin‐derived mineralogy (Bristow et al., 2018; Rampe et al., 2020) from select drill targets in these members. These members of the Murray formation were selected because they are fine‐grained in nature and lie below (Figure S1) the Vera Rubin ridge (sub‐VRR) region, which is interpreted as a significantly altered diagenetic front within the Gale crater stratigraphy (e.g., Fraeman et al., 2020; Rampe et al., 2020). It is important to note here that the CheMin instrument is not deployed every time the APXS instrument is, thus drill target mineralogy included here (Figure 1) only represents a subset of the geochemical analyses displayed in subsequent figures.

Terrestrial geochemical reference values from the literature were gathered for soil weathering profiles and fluvial sediments in basaltic terrains (Caner et al., 2014; Craig & Loughnan, 1964; De Carlo et al., 2005; Gibson et al., 1983; Ma et al., 2007; H. W. Nesbitt & Wilson, 1992; Pokrovsky et al., 2005; Porder et al., 2007; Rasmussen et al., 2010; Yesavage et al., 2015), all of which are detailed in supplementary text S1 and Table S3. Data sets from the literature were selected based on the availability of geochemical data coupled with well‐documented information on the climate of each particular study site and with the goal of spanning a significant climatic range. We use both weathering profiles and fluvial sediments because weathering profiles provide a way to assess chemical weathering at the sediment source; fluvial sediments provide information on provenance mixing and any alteration along the transportation pathway from source‐to‐sink. Geochemical and mineralogical data compiled from M. T. Thorpe et al. (2019) come from study sites in the Hvítá S catchment in Iceland, which is a 6,714‐km^2^ watershed situated almost entirely in Pleistocene basalts with a tholeiitic composition and a mineral assemblage dominated by plagioclase, pyroxene, primary glass, and minor olivine (Figure S1). Geochemical and mineralogical data compiled from M. T. Thorpe and Hurowitz (2020) come from sediments collected in the Clearwater River watershed in Idaho, USA. These sediments are primarily sourced from the Grande Ronde member of the CRB group, which is chiefly composed of plagioclase and pyroxene, with trace amounts of olivine and magnetite (Reidel et al., 1989) and covers between 62% and 85% of the watersheds investigated by M. T. Thorpe and Hurowitz (2020) (Figure S1). Both of these studies focused on an examination of the geochemistry and mineralogy of sediment sources, and fluvial sediments transported from their source regions to depositional sites downstream, while also exploring the effects of grain size sorting during transport.

## 3 Data Analysis

In our suite of terrestrial samples, when grain sizes were reported, we only consider the sediment <63 μm, which includes the Wentworth (1922) silt (63–4 μm) and clay (<4 μm) grain size classes, collectively referred to as “mud.” We compare those samples with data collected from martian “mudstones,” broadly defined in Siebach et al. (2017) as sedimentary rocks with grain sizes below the resolution of the Mars Hand Lens Imager on the Curiosity rover, which has 30 μm/pixel resolution at a 5‐cm camera standoff from the martian surface, enabling grains larger than ∼50–100 μm in diameter to be resolved.

Mineralogical comparisons between terrestrial mud‐sized sediments from Iceland and Idaho and martian mudstones are based on mineral abundances derived from X‐ray diffraction (XRD) patterns (Figures 1a and 1b). Direct comparison of the XRD patterns from Gale crater mudstones and Icelandic muds reveal first‐order similarities, including (1) low angle two‐theta reflections for smectite clay minerals, (2) an elevated background from ∼19° to 35° two‐theta that is associated with scatter from one or more poorly ordered and/or X‐ray amorphous phase(s), and (3) abundant primary mafic minerals, chiefly plagioclase and pyroxene. While the Idaho mud XRD pattern similarly reveals the presence of smectite, the sample has less abundant mafic minerals and contains quartz and mica; the latter interpreted by M. T. Thorpe and Hurowitz (2020) to reflect a more complex provenance and weathering history. The mineral abundances of the Gale crater mudstones and Icelandic silt and clay display similar proportions of primary silicates, diagenetically immature clays (e.g., smectite), and X‐ray amorphous material, whereas the Idaho mud is significantly enriched in multiple clay mineral phases by comparison (Figure 1b).

**Figure 2:**
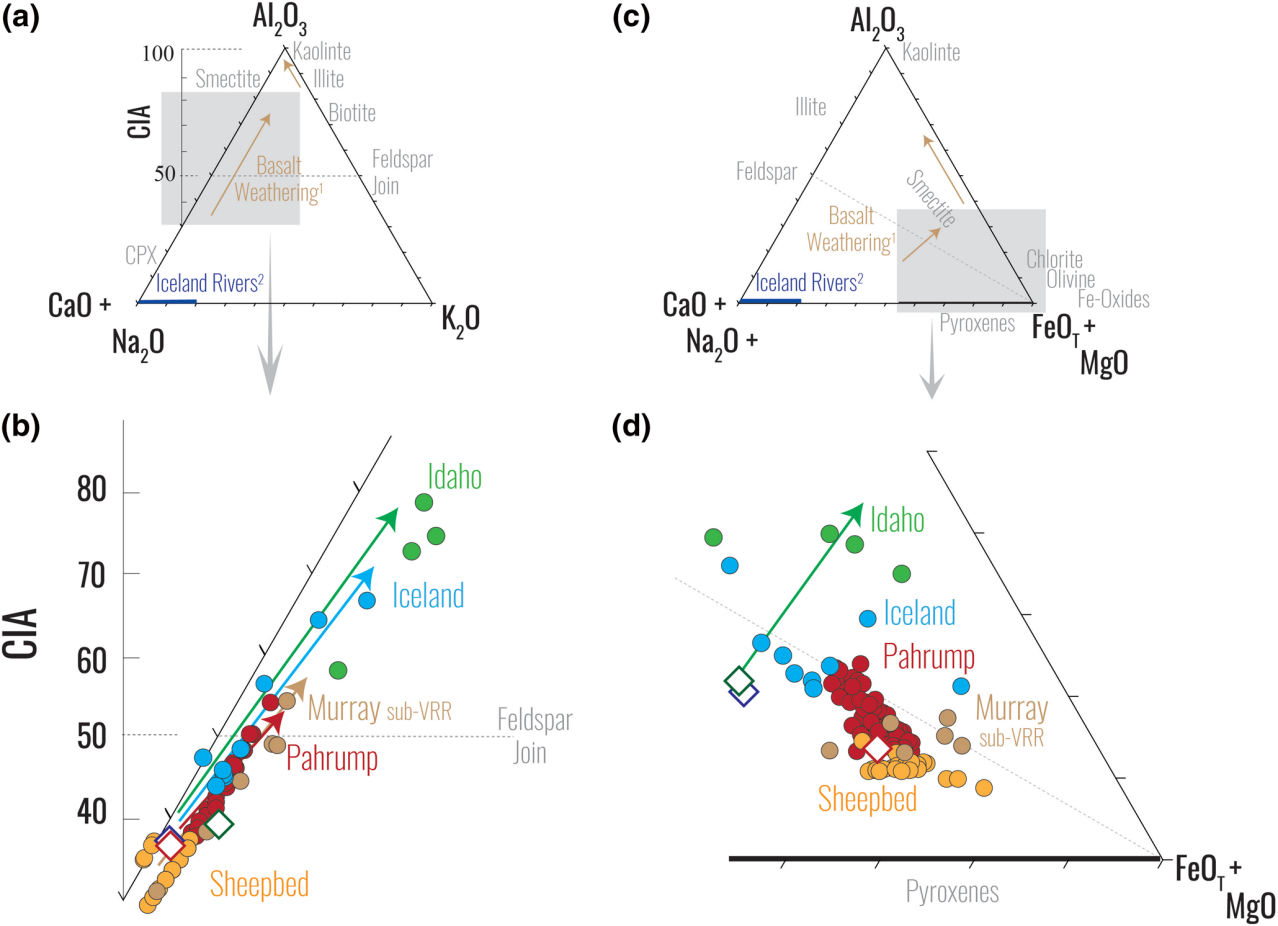
Geochemical data plotted in molar ratios on an A–CN–K (a and b) and an A–CNK–FM (c and d) diagram for the mud‐sized terrestrial sediments and mudstones from Gale crater, Mars (circles), as well as terrestrial source rock compositions from Iceland and Idaho and an estimate for the martian crust (diamonds). While the Sheepbed mudstones display little to no change relative to the martian crust, the Murray mudstones display evidence for chemical alteration. ^1^Basalt weathering trends: H. W. Nesbitt and Wilson (1992). ^2^Icelandic Waters: Gislason et al. (1996).

Terrestrial mud‐sized sediments from Iceland and Idaho and martian mudstones are plotted on an A–CN–K plot (Figures 2a and 2b), with the molar ratios of Al_2_O_3_, (CaO + Na_2_O), and K_2_O at the apices of a ternary plot. On this ternary, feldspar (as well as clinopyroxene and glass) dissolution and the production of secondary weathering products result in weathering trends from Ca‐rich, Na‐rich, and K‐rich rocks and minerals toward alumina‐rich secondary products (H. W. Nesbitt & Young, 1984). In subaerial weathering of basalts, these trends parallel the A–CN boundary that extends from basalt compositions enriched in CaO and Na_2_O to secondary weathering products that are Al_2_O_3_ enriched (e.g., H. W. Nesbitt, 2003).

We note here that, for all terrestrial and martian samples, we use as‐measured CaO abundances in the calculation of CIA values. CIA is generally corrected so that only the calcium that resides in the silicate fraction is considered, thereby excluding nonsilicate minerals which add Ca^2+^ back into the sample (esp., calcite, apatite), which would otherwise make the silicate fraction appear less chemically weathered than it is (McLennan et al., 1993; H. Nesbitt & Young, 1982; H. W. Nesbitt & Wilson, 1992). However, this correction is challenging for the martian samples using bulk APXS data and so it is not applied to any samples here, for reasons elaborated on in the supplementary text S2, and well summarized in McLennan et al. (2014). We selected the Sheepbed and Pahrump Hills members for the main comparisons discussed here because those two units show limited ranges of CIA values that are consistent with minimal affects from added salts. The net effect of using total CaO instead of CaO* is that our CIA values are all minimum estimates that reflect a conservative estimate of the extent of chemical weathering. For an alternate view of the CaO* correction on Mars, see Schieber et al. (2017).

In addition to the A–CN–K diagram, a second ternary diagram plots molar Al_2_O_3_, (CaO + Na_2_O + K_2_O), and (FeO_T_ + MgO) at the apices, forming the A–CNK–FM diagram (Figures 2c and 2d); the incorporation of Fe and Mg makes this diagram particularly useful in the evaluation of major element mobility in basalt (H. W. Nesbitt & Wilson, 1992). Chemical weathering trends on the A–CNK–FM diagram are indicated by a trend that runs subparallel to the A–CNK margin with a trajectory toward the A–FM boundary, resulting from preferential removal of CaO, Na_2_O, K_2_O, and MgO and retention of Al_2_O_3_ and FeO_T_ in rocks undergoing oxidative chemical weathering.

**Figure 3:**
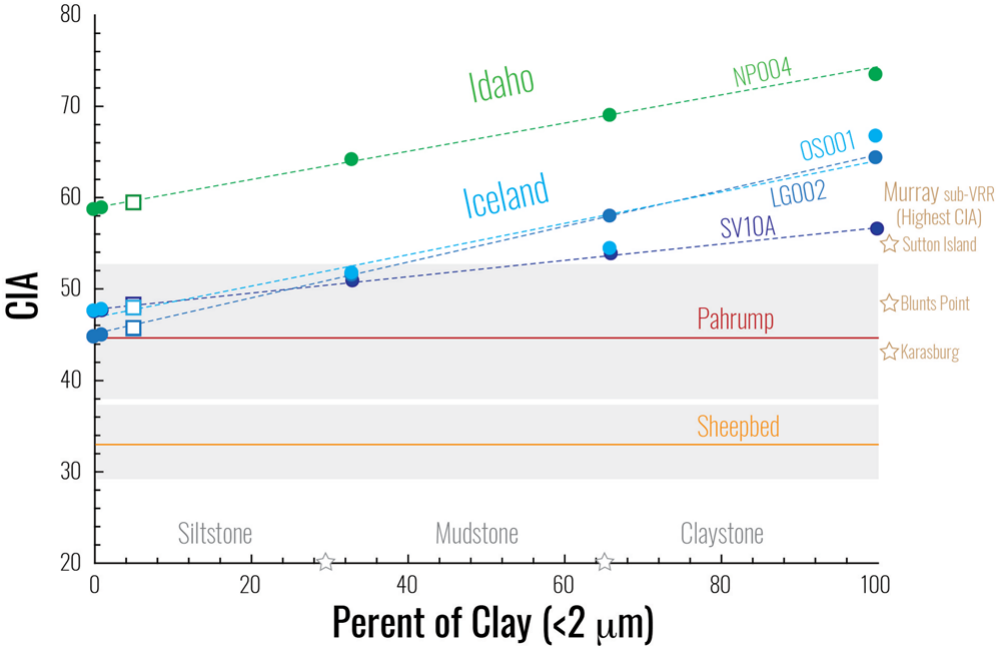
Chemical index of alteration (CIA) values plotted against linear mixing models for mudrock composition based on the percent of the clay size fraction. Terrestrial analog sample IDs are next to the linear models and bulk CIA values (open squares) are calculated for a mudrock with approximately 5 wt% of <2 μm particles. CIA ranges (gray box) and averages (solid lines) are shown for the Sheepbed and Pahrump mudstones, while mudstones from higher in the Murray stratigraphy are labeled with the highest CIA value plotted as a point (star) on the right side of the diagram. On the abscissa, mudrock nomenclature was adopted from Folk (1980).

In order to make a more direct comparison, it is important to note that the terrestrial mud samples from M. T. Thorpe et al. (2019) and M. T. Thorpe and Hurowitz (2020) were physically separated and sieved, with the masses of various grain size fractions determined in the laboratory (Supporting Information S3). In comparison, the martian mudstones are naturally sorted mixtures that include grain sizes ranging from clay to silt in uncertain relative proportions. Thus, we constructed a linear mixing model to derive the CIA values of mixtures of various proportions of clay‐sized and silt‐sized terrestrial sediments (Table S4). This calculation reveals that CIA values are linearly related (Figure 3) to the ratio of clay to silt for both terrestrial sediment suites.

**Figure 4:**
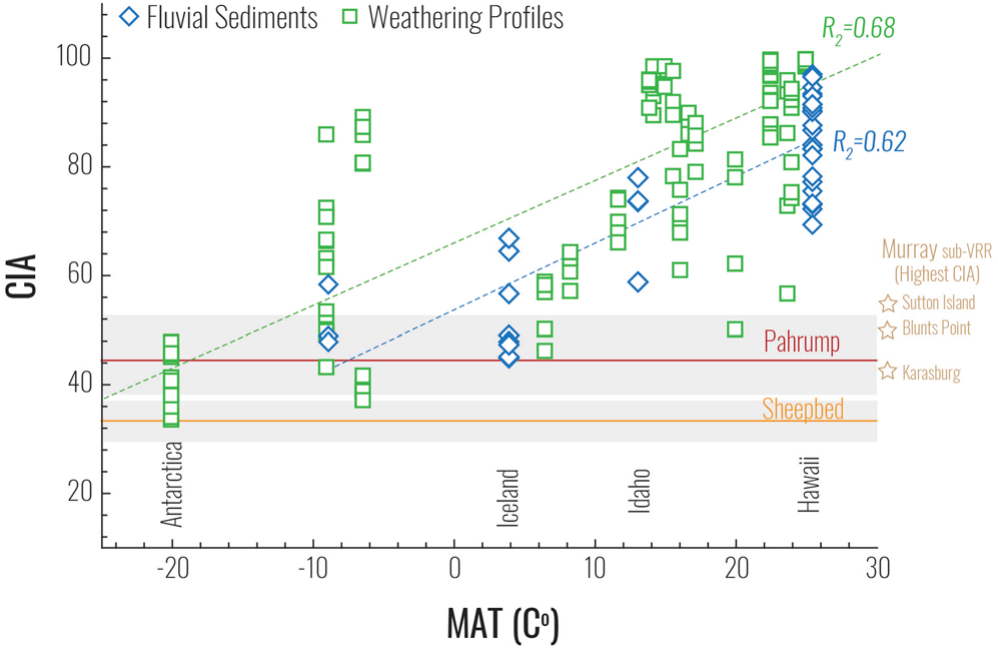
Chemical index of alteration (CIA) values plotted against mean annual temperature (MAT) compiled from the literature in comparison to CIA ranges (gray box) and averages (solid lines) Sheepbed and Pahrump mudstones, while mudstones from higher in the Murray stratigraphy are labeled with the highest CIA value plotted as a point (star) on the right side of the diagram.

Lastly, to better understand the degree to which the chemical composition of sediments in mafic environments can be used to make further quantitative climatologic inferences, CIA is compared to the climate variables mean annual temperature (MAT, Figure 4) and mean annual precipitation (MAP, Figure S3) for a wider suite of published analyses from basaltic weathering profiles and fluvial sediments (see Section 2). From these data sets, we note that MAP and MAT are not necessarily correlated since MAP can vary significantly across a range of MAT values (Figure S4), with temperature controlling whether most of the precipitation falls as rain or snow. In this study, we are interested in exploring whether CIA in basaltic sediments, as a paleoclimate indicator, exhibits any first‐order dependence on either (or both) of these climate variables. We also explore other variables that may influence CIA, including the geological age of the source rocks, elevation of weathering profiles, grain size of fluvial sediments, depositional site of fluvial sediments, and distance fluvial sediments are transported from the source regions (Figures S5–S9).

## 4 Results and Discussion

From our literature compilation, MAT and grain size appear to be the major variables most strongly correlated with CIA variations in martian analog terrains. Therefore, directly comparing similar grain sizes across planets allows us to effectively understand the impact of temperature on CIA. Terrestrial mud‐sized materials from analog environments and martian mudstones display not only remarkable similarities but also distinct differences that help constrain the paleoclimate in ancient Gale crater. We first focus on exploring the similarities and differences between the martian mudstones and the subset of terrestrial sediments from Iceland and Idaho because these data sets have well‐documented mineralogy, geochemistry, and sedimentology. The full suites of terrestrial materials are then used to provide improved constraints on the paleoclimate influencing chemical weathering in Gale crater.

### 4.1 Mineralogy

Mineralogical differences between the terrestrial sediments from Iceland and Idaho suggest that mineralogy may provide clues to the type of climate influencing chemical weathering. Comparing the mineralogy from this subset of terrestrial analog sediments with data returned from *Curiosity*, we observe strong similarities between mineral identities and abundances when comparing martian mudstones and sediments from Iceland. The XRD patterns from these mud‐sized sediments from Iceland reveal primary igneous minerals (i.e., plagioclase, pyroxene, and olivine), smectite as the only clay mineral identified, and an elevated background from X‐ray amorphous scattering. M. T. Thorpe et al. (2019) identified the smectitic phase as a dioctahedral smectite and concluded that the X‐ray amorphous component was at least partially derived from chemical alteration. Additional high‐resolution analysis of X‐ray amorphous materials in Icelandic sediments (M. T. Thorpe et al., 2020) and mafic sediments from the continental United States (Rampe, Horgan, et al., 2017) as well as other studies modeling X‐ray amorphous composition for other mafic locales throughout the world (Smith et al., 2018) collectively demonstrates that this component is a complex and intimate mixture of primary igneous glasses with secondary phyllosilicates and multiple nanophase materials.

Similarly, the mineralogy of the Gale crater mudstones is broadly characterized by a mixture of primary igneous minerals, smectite phyllosilicates, and a significant contribution from an X‐ray amorphous component. The clay minerals in the Gale crater mudstones are largely identified as smectites; however, the smectitic phase is a trioctahedral saponite in the Sheepbed mudstones (Vaniman et al., 2014) and predominately dioctahedral Fe‐bearing smectites increasing upsection in the Murray formation (Bristow et al., 2018). Determinations of the composition of the martian X‐ray amorphous component have relied on mass balance calculations that have shown it to be compositionally variable throughout the Gale crater stratigraphy. Published models indicate a diverse mixture of amorphous phases with varying proportions of primary igneous glass, nanophase ferric oxides (e.g., ferrihydrite), Fe/Si bearing amorphous materials (e.g., hisingerite), and amorphous sulfates (e.g., Achilles et al., 2020; Dehouck et al., 2014; Rampe, Ming, et al., 2017). While there are differences in the identification of minor mineral phases between the mudstones from Gale crater and the sediments from Iceland, the relative proportions of primary minerals, secondary clay minerals, and X‐ray amorphous materials are qualitatively comparable (Figure 1b). Furthermore, it is also important to note that in both the Icelandic sediments and the Gale crater mudstones investigated here, smectites are the only clay minerals observed and both sample suites contain a diverse X‐ray amorphous component. These first‐order similarities demonstrate that weathering and transport processes in a cold fluvial source‐to‐sink system have had similar effects on the overall mineralogy of these suites of basaltic sediments despite likely differences in atmospheric properties and the influence of biologically mediated processes on Earth. We note the enigmatic observation that there are significant similarities between weathering products in a suite of modern terrestrial sediments and ancient (>3.5 Ga) sedimentary rocks on Mars. This observation defies explanation with our current terrestrial reference frame, apparently requiring little or no diagenetic maturation in the martian rocks despite rover observations indicating burial of these sedimentary rocks to ∼1–3 km depth (Caswell & Milliken, 2017; Lewis et al., 2019), where transformation of smectite to illite/chlorite and amorphous silica to quartz is reasonably expected (e.g., Hower et al., 1976). Although minor amounts of chlorite have been identified from orbit in other locales (Sun & Milliken, 2015), juvenile alteration products (e.g., smectite and hydrated silica) still dominate the secondary mineral assemblage (e.g., Tosca & Knoll, 2009), and thus the diagenetic history of sedimentary rocks on Mars remains an outstanding issue.

In contrast, the XRD pattern of the mud‐size sediment from Idaho contains relatively low abundances of primary mafic minerals, and the secondary minerals identified are distinct from the Icelandic mud‐sized sediments and inconsistent with what has been observed in Gale crater mudstones from the standpoint of mineral identity and diagenetic maturity. Moreover, the differences in the secondary mineralogy, specifically the clay minerals phases present in the sediments from Idaho, highlight how the products of chemical weathering in a warmer climate are largely inconsistent with the observed mineralogy of Gale crater mudstones. For example, sediments from Idaho display a diverse suite of clay mineral phases, with multiple smectites, kaolinite, and illite all identified in this sample suite. Collectively, this clay mineral assemblage was interpreted to have formed as a results of incipient alteration (smectites), advanced weathering (kaolinites), and eolian input (illite) (M. T. Thorpe & Hurowitz, 2020), illustrating a fundamentally more advanced stage in sedimentation when compared to the Gale crater mudstones. The mineralogical data from Idaho prove useful in unraveling other sedimentary trends, including mixing and sorting. In the sediments from Idaho, quartz and potassium feldspar are also abundant, neither of which are derived from basaltic sources in the sediment catchment. M. T. Thorpe and Hurowitz (2020) concluded that these felsic mineral phases were derived from mixing with sediment derived from nonmafic sources present at relatively low abundance in the watershed, with the highest abundance of felsic material in sand‐sized to silt‐sized samples. Interestingly, Gale crater sedimentary deposits also contain variable amounts of felsic detritus, especially K‐spar and lesser quartz. Indeed, a sandstone target (“Windjana”) from the Kimberly formation in the Bradbury group contains 21 wt% potassium feldspar, which led Treiman et al. (2016) to suggest that this coarser grained sedimentary rock had a mixed provenance with an evolved volcanic component. If mafic‐felsic provenance mixing was an active process in the sedimentary system of Gale crater, then by analogy with Idaho, felsic material may be expected to be most abundant in the sand to silt fraction.

### 4.2 Mars, Iceland, and Idaho Weathering and Grain Size Trends

Comparison between martian mudstones and terrestrial mud‐sized materials from Iceland and Idaho indicates that uncorrected (i.e., minimum) CIA values in the Sheepbed member mudstone (average = 33.8) are significantly lower than those in sediments from Iceland (average = 51.6) or from Idaho (average = 70.9), whereas the Pahrump Hills values (average = 44.5) as well as the highest CIA values for mudstones higher in the Murray formation (i.e., sub‐VRR) are closer to the Icelandic range (Figure 2). The Sheepbed mudstones display low CIA values that are nearly identical to the martian crust (36.9). These values cluster on the A–CN–K and A–CNK–FM diagrams (Figure 2) and imply little to no element mobilization or fractionation from basalt source to sediment sink, consistent with a cold and/or arid paleoclimate (McLennan et al., 2014). In contrast, the Pahrump Hills mudstones display higher CIA values and plot along a trajectory that indicates chemical weathering has influenced the composition of these rocks (Figure 2). While the overall compositional trend for the Pahrump Hills mudstones on the A–CNK–FM diagram (Figure 2) is not fully explained by the normal basalt weathering array, the weathering component is similar to the spread observed in the Icelandic sediments. This shift to higher CIA values and the observable weathering trend values relative to the Sheepbed member is consistent with a transition toward more clement conditions (Hurowitz et al., 2017).

*Curiosity* has continued its exploration in the Murray formation, covering >200 more meters of stratigraphy since leaving the Pahrump Hills formation as of the writing of this manuscript. However, most of the Murray members above Pahrump Hills do not show consistent uncorrected CIA values, reflecting significant and variable addition of secondary salts, so those members require a different CIA correction approach than we have taken here (e.g., Siebach & McLennan, 2018 and *see also* supporting information text S2). For reference, because added secondary salts would only serve to lower CIA values in those Murray members, we plot the highest uncorrected CIA value from the mudstone‐dominated formations between Pahrump Hills and the diagenetically altered Vera Rubin ridge. These are still minimum CIA values for the targets considered. Mudstones from the Karasburg and Blunts Point members of the Murray formation are near the average Pahrump Hills mudstone value, while the Sutton Island member contains the highest uncorrected CIA value included here, indicating more open system weathering upsection. The open system weathering in sub‐VRR Murray units is also displayed on the A–CNK–FM diagram through a scatter around the feldspar–FM tie line in a manner similar to what is observed in the Icelandic sediments. This simplified addition of the highest uncorrected CIA value from the rest of the Murray mudstones demonstrates subtle changes in the paleoclimate throughout the Murray, with CIA values that closely resemble the Icelandic range.

The finding of increasing CIA values upsection in the Murray mudstone has been supported by other approaches in the literature; geochemical data from the CheCam instrument onboard *Curiosity* also indicate that mudstones in the Sutton Island member represent the most altered sedimentary rocks in the Gale crater stratigraphy sub‐VRR (Mangold et al., 2019), and open system weathering products predominating in the upper Murray (Bristow et al., 2018). Mangold et al. (2019) utilize the CheCam instrument onboard *Curiosity*, which operates with a smaller analytical spot size than APXS (∼300 microns vs. 1.7 cm), they were able to identify analytical points that appeared to have been diagenetically influenced and then average the remaining points to approximate a bulk unaltered mudstone composition. Calculations of CIA on a data set that had diagenetically influenced analyses removed (Mangold et al., 2019) indicate a smaller range of CIA values than observed in the APXS data set, and a maximum CIA in the Sutton Island member that is higher than the highest APXS derived CIA reported here. Both approaches provide strong evidence for chemical weathering in an open system during the sedimentation in the Murray catchment, although uncorrected bulk APXS values provide a more direct baseline for paleoclimate comparisons with terrestrial bulk composition data.

This open system weathering is also displayed on the A–CN–K diagram and consistent with both the normal terrestrial weathering array and Pahrump Hills trend (Figure 2a). On the A–CNK–FM diagram, the weathering array for Murray mudstones below the VRR is inconsistent with the expected basalt weathering trend but similar to the previously described scatter displayed by the Pahrump Hills mudstones and Icelandic sediments (Figure 2b), indicating that paleoclimate changes recorded by the Murray formation above Pahrump Hills are subtle (at least at the scale of resolution of APXS) and broadly similar to what is displayed by sediments from Iceland.

In the terrestrial study sites of Iceland and Idaho, M. T. Thorpe et al. (2019) and M. T. Thorpe and Hurowitz (2020) found that the degree of chemical weathering increases as grain size decreases, resulting in the most chemically altered material preferentially concentrated into the finest grain size, that is, the clay size fraction (<2 μm) of fluvial sediments (Figure S5). However, even with this sediment sorting mechanism, MAT plays a significant role in the extent of alteration, as the fine‐grained fraction of sediment from Idaho has higher CIA values compared to sediment from Iceland (Figure 3). Together, these two variables demonstrate that the climate is best recorded the finest size fraction for silicate‐dominated systems. It is also important to note when making planetary comparisons that the processes controlling this enrichment of altered material in the <2‐μm sediment are both intrinsic, as weathering reduces particles size and creates a higher volume of surface area to weather, and extrinsic, as sediment sorting during transport concentrates particles of the same size and density. When we then compare the CIA values of the Sheepbed mudstones to the calculated CIA ranges of terrestrial basaltic mud, the Sheepbed CIA values are lower than any calculated mud composition, regardless of the grain size, either in Iceland or Idaho (Figure 3). This reinforces the interpretation that during the formation of Sheepbed mudstones, the paleoclimate did not promote chemical alteration and that extremely cold and arid conditions prevailed, allowing physical weathering rates to outstrip chemical weathering rates in the source terrains. In contrast, the Pahrump Hills mudstone CIA range of 38.1–52.6 extends into the modeled silt to mud CIA range for samples from Iceland (Figure 3) but does not overlap with the range of CIA values in sediments from Idaho. Specifically, sediments from Iceland with 44% or less of <2 μm clay exhibit similar CIA values to the Pahrump Hills. CIA values in the Sutton Island member also fall within the range of CIA values exhibited by mud‐sized (<63 μm) sediment from Iceland. One could interpret these relationships in one of two ways: if we assume that all Murray formation mudstones possess relatively homogeneous and invariant grain size characteristics, then those grains exhibit bulk chemical properties consistent with an amelioration in climate toward warmer conditions upsection, and within the range of CIA values exhibited by Icelandic sediment. Alternatively, climate conditions could have been relatively stable during deposition of Murray formation mudstones, with the mudstones becoming systematically finer‐grained upsection. The observations needed to unambiguously distinguish between these hypotheses are beyond the resolution limits of the *Curiosity* instrument payload and probably require a thin section to resolve. However, we are unaware of any sedimentological or stratigraphic relationships that strongly support the latter possibility and suggest that the former hypothesis represents a reasonable interpretation given available constraints.

### 4.3 CIA and Climate Variables

Our literature compilation of basaltic weathering profiles and fluvial sediment geochemistry spans a range of temperature and precipitation values, allowing us to take our comparative reference frame from Iceland and Idaho a step further and examine the effects of climate variables on sediment and sediment source geochemistry in mafic terrains. As shown in Figure 4, MAT appears to exert the most obvious direct control on CIA, especially when compared to other environmental variables. For example, MAP exhibits only a scattered positive linear relationship with CIA (Figure S3). All but one sample with MAP > 1,340 mm/year have CIA values exceeding 73, suggesting that significant annual precipitation enhances chemical weathering. However, environments with lower MAP span a significant range of CIA values, with samples ranging from CIA values of ∼36 to >80, indicating that temperature, perhaps combined with some of the other variables described below, exerts stronger controls on CIA values in drier environments.

Elevation is positively correlated with higher CIA values in stream sediments from Hawaii; however, weathering profiles in the Cascade Range demonstrate an inverse correlation mainly because higher elevation profiles are also exceedingly cold (Figure S6). For fluvial sediments, the distance sediments travel from the source regions is positively correlated with CIA (Figure S7). Similarly, depositional sites within the stream profile (e.g., stream bed vs. flood plains) also contribute to CIA variations, hinting toward continued alteration in temporary storage sites and/or facies‐dependent grain size (and CIA) fractionation (Figure S8). There is also an offset of weathering profiles toward slightly higher CIA values compared to fluvial sediments, suggesting a difference in physical versus chemical weathering between these sample suites, as discussed in supporting information text S4. Finally, geological age of the source rock appears to have little impact on the CIA values of both fluvial sediments and weathering profiles (Figure S9). Even the youngest samples in our data set have elevated CIA values, suggesting even weathering profiles ∼10 ka have ample time for the onset of advanced chemical weathering given appropriate climate conditions.

Returning to Figure 4, we suggest that MAT and CIA are clearly the most strongly correlated variables, expressing a positive linear relationship with each other, such that warmer temperatures produce higher CIA values in mafic terrains, as expected. When terrestrial CIA values and mineralogy of fine‐grained sediments are compared with mudstones from Gale crater, we can place improved constraints on the paleoclimate history of Gale crater. It is important to note here that because the calculated CIA values are uncorrected for any nonsilicate or diagenetic cation addition and represent a chemical weathering minimum, that the climate comparisons with Earth detailed below should also represent a baseline climate from which warmer deviations could be allowed. With that in mind, we suggest that the type of climate inferred from the composition of the Sheepbed mudstones is consistent with average annual temperatures below freezing and, while MAP totals are difficult to extrapolate, it seems reasonable to infer that MAP may have been below 500 mm. We suggest that the Sheepbed formation may have formed during a potentially brief climate optimum just above freezing, when water could flow. In contrast, we suggest a climate similar to that of Iceland, with MAT from ∼2 to 5.7°C and MAP ranging up to ∼1,600 mm/year, serves as a reasonable analog for the paleoclimate during the deposition of the Pahrump Hills member of the Murray formation. Similarly, mudstones from the Karasburg and Blunts Point and Sutton Island members well explained by Icelandic‐like conditions. In addition to these proposed climate scenarios, the low CIA values and the preservation of primary igneous minerals in both Icelandic fluvial sediments and Gale crater mudstones suggest a sedimentation history strongly influenced by physical weathering rather than chemical weathering in the source terrains.

### 4.4 Implications for Mars

The geochemical and mineralogical characteristics of basaltic sediment in watersheds with varying climates on Earth provide a reference frame for understanding the type of climate present during the time period when the Gale crater watershed was active. While there is no perfect terrestrial analog for the Gale crater watershed, this ancient environment is consistent with a subpolar to polar climate that was variably icy/wet, in accordance with the Köppen classification scheme used on Earth (Kottek et al., 2006). Comparison of the geochemistry of the Sheepbed mudstone to that of terrestrial analog materials indicates that environmental conditions could have been as cold as those measured where weathering profiles are presently developing in Antarctica (e.g., −20°C) and as wet as climates where MAP reaches 385 mm/year (e.g., Svalbard in our terrestrial analog suite). For Sheepbed member mudstones at the base of the Gale crater section, incipient weathering occurred in the source terrains, producing minimally altered detritus that was eroded and carried via rivers and streams to depositional sites downstream. Fine‐grained incipient alteration products and fine‐grained mafic mineral detritus (i.e., olivine and pyroxene) were both concentrated into the mud‐sized sediment. Following deposition, this sediment was subjected to diagenetic alteration, as proposed by Bristow et al. (2015) and McLennan et al. (2014), resulting in the formation of neoformed trioctahedral smectites that are different than the smectitic phase(s) identified in fluvial sediments from other terrestrial study sites. As the paleoclimate shifted during the formation of the Pahrump Hills and upper Murray mudstones to a subpolar climate, reflecting more Icelandic‐like conditions, the nature of the clay minerals also shifted. Previous work by Bristow et al. (2018) attributes the observed transition to a dioctahedral smectite dominated mineralogy as resulting from authigenic conditions in the lake shifting from a closed to an open system. The Icelandic sediments in M. T. Thorpe et al. (2019) provide an alternative explanation for this shift: their observations indicate that that in fluvial systems in subpolar climates on Earth can also produce dioctahedral smectites as the sole phyllosilicate phase, indicating that weathering profiles in the source terrains (and/or chemical weathering throughout the transportation pathway) are also plausible analogs for the sources of clay minerals in the Murray mudstones. Both scenarios would result in the transport of partially altered clastic detritus and produce muds downstream with higher CIA values. Overall, the geochemistry and mineralogy of fine‐grained rocks in Gale crater record evidence of a paleoclimate that shifted from Antarctic‐like conditions to Icelandic‐like conditions, and conditions on Mars may well have become more clement in later periods, a hypothesis Curiosity may be able to test as it continues to traverse into younger terrains. These results imply a dynamic Hesperian climate in the Gale crater region and provide geochemical and mineralogical constraints for evaluating the paleoclimate history from sedimentary strata at future Mars rover landing sites (e.g., Jezero crater).

## 5 Conclusions

The direct comparison between X‐ray diffraction patterns and mineral abundances from basaltic sediments on Earth and 3.5 Ga basaltic mudstones on Mars demonstrates that both cold environments produce similar phases including abundant primary mafic igneous minerals accompanied by smectitic clay and X‐ray amorphous materials. Lithification and diagenesis on Mars did not significantly change the phase assemblage compared to Iceland. Furthermore, the CIA values of terrestrial sediments from basaltic watersheds can be reliably used as a climate proxy and extended to Mars, with MAT exerting a controlling influence on CIA. Our comparison of the geochemistry of martian and terrestrial sediment indicates that the baseline for the paleoclimate around Gale crater shifted from a polar climate (e.g., Antarctic‐like) during deposition of the Sheepbed member of the Bradbury Group to a subpolar climate (e.g., Icelandic like) during deposition of the Pahrump Hills member of the Mount Sharp Group.

## Supporting information

Supporting Information S1

## Data Availability

Data sets for this research are included in this paper and tables of reference values are compiled its supplementary information files. For martian data sets, data are available from McLennan et al. (2014), Hurowitz et al. (2017), Vaniman et al. (2014), Rampe, Ming, et al. (2017), Bristow et al. (2018), and Berger et al. (2020). Terrestrial data sets are compiled in the supporting information text S1, and data are available from Gibson et al. (1983), Craig and Loughnan (1964), H. W. Nesbitt and Wilson (1992), Pokrovsky et al. (2005), De Carlo et al. (2005), Porder et al. (2007), Ma et al. (2007), Rasmussen et al. (2010), Caner et al. (2014), Yesavage et al. (2015), M. T. Thorpe et al. (2019), and M. T. Thorpe and Hurowitz (2020). The data sets for this work are also archived on a FAIR repository and can be accessed at Thorpe (2020).
