# Supplementary material for: Source‐to‐Sink Terrestrial Analogs for the Paleoenvironment of Gale Crater, Mars
Source: J Geophys Res Planets. 2021 Feb 18;126(2):e2020JE006530. doi: 10.1029/2020JE006530 (PMC7988529; doi:10.1029/2020JE006530)
Supplement: Supplementary file 1 — Supporting Information S1 [file JGRE-126-e2020JE006530-s001.docx]

*Journal of Geophysical Research-Planets*

Supporting Information for

**Source-to-Sink Terrestrial Analogs for the Paleoenvironment of Gale Crater, Mars**

Michael T. Thorpe^1,2*^, Joel A. Hurowitz^3^, and Kirsten L. Siebach^2^

^1^Department of Earth, Environmental and Planetary Sciences, Rice University, 6100 Main Street

Houston, TX 77005 USA

^2^NASA Postdoctoral Program, NASA Johnson Space Center, Houston, TX 77058, USA

^3^Department of Geosciences, State University of New York at Stony Brook, Stony Brook, NY 11794-2100, USA

*Corresponding author: Michael T. Thorpe; [michael.thorpe@rice.edu](mailto:michael.thorpe@rice.edu); [michael.t.thorpe@nasa.gov](mailto:michael.t.thorpe@nasa.gov)

**Contents of this file**

**Text s1:** *Additional References and Descriptions*

**Text s2:** *Discussion of CaO**

**Text s3:** *Weighted Average of Terrestrial Muds and Hypothetical Mudrock*

**Text s4**: *Weathering Profiles vs. Fluvial Sediments*

**Supplementary Figure S1**. Martian and terrestrial study sites

**Supplementary Figure S2**. A stratigraphic column of Bradbury and Mount Sharp group

**Supplementary Figure S3**. Chemical index of alteration (CIA) values plotted against mean annual precipitation (MAP)

**Supplementary Figure S4**. Mean annual precipitation (MAP) plotted against mean annual temperature (MAT)

**Supplementary Figure S5**. Chemical Index of Alteration (CIA) values plotted again grain size

**Supplementary Figure S6**. Chemical Index of Alteration (CIA) values plotted against elevation

**Supplementary Figure S7**. CIA vs. distance from the source

**Supplementary Figure S8**. Chemical Index of Alteration (CIA) values versus depositional site

**Supplementary Figure S9**. Chemical Index of Alteration (CIA) values versus source rock age

**Table s1**. Mars Geochemistry

**Table s2**. Martian and Terrestrial Mineralogy

**Table s3**. Terrestrial geochemistry

**Table s4**. Mudstone models

**Table s5. Depositional Site CIA values**

| **Text s1:** *Additional References and Descriptions* | |
| --- | --- |
| **Reference** | **Notes** |
| *Rasmussen et al*., (2010) | Basalt weathering and pedogensis in Cascade Range, California, USA. Geological age assumed from the oldest age of the period identified in the citation. |
| *Pokrovsky et al*., (2005) | Basalt weathering profile and fluvial sediment from Central Siberia under permafrost conditions. The annual precipitation varies from 400 to 450 mm in the eastern part of the plateau to 500 to 550 mm in the western part. In this work we choose 475 mm/yr as the representative value. Weathering profile and fluvial sediment CIA calculations excluded the <2 mm coarse grained sediments from this reference and used the <1 μm and 1 to 50 μm grain size fractions. |
| *Nesbitt and Wilson*, (1992) | Weathering profile of the Baynton Basalts northwest of Melbourne, Australia. Daytime summer temperatures range from 30 to 38°C and daytime winter temperature range from 10 to 17°C. In this work, we approximated a MAT of 24°C. Geological age assumed from the oldest age of the period identified in the citation. CIA values were excluded for the interior rock samples (A-1 and all of B-1) as well as samples still along the core of the source basalt (A-2, A-3, A-4). We focused here on the soil profile. |
| *Ma et al*., (2007) | Weathering profile of Neogene basalts in northern Hainan Island, South China. CIA values were excluded for the parent rocks. |
| *Yesavage et al*., (2015) | Soils from Quaternary basaltic flows at the Sverrefjell volcano in Svalbard. Only the clay fraction is plotted. |
| *Caner et al*., (2014) | Basalt weathering and soil formation in southern Brazil. |
| *Gibson et al*., (1983) | Weathering soils in the Dry Valleys of Antarctica.  Precipitation is all in the form of snow and ranges from 5 to 10 cm/yr. In this work, we took the median of 7.5 cm/yr. |
| *Porder et al*., (2007) | Soil formation across precipitation gradient in Hawaiian Islands. CIA values were calculated for the soils from the B and C horizon. The maximum CIA value was used from each profile in order to display the greatest effect of climate on alteration. the MAT values were determined from the general coordinates for field locations on Figure 1 of *Porder et al*., 2007 and the Geography Department’s climate database from University of Hawaii. Please see: http://climate.geography.hawaii.edu/interactivemap.html and the following reference:  *^1^Giambelluca, T.W., X. Shuai, M.L. Barnes, R.J. Alliss, R.J. Longman, T. Miura, Q. Chen, A.G. Frazier, R.G. Mudd, L. Cuo, and A.D. Businger*. 2014. Evapotranspiration of Hawai‘i. Final report submitted to the U.S. Army Corps of Engineers—Honolulu District, and the Commission on Water Resource Management, State of Hawai‘i. |
| *Thorpe and Hurowitz*, (2020) | Fluvial basaltic sediments from Idaho. Geological age assumed from the oldest age of the period identified in the citation. Unconsolidated sediment samples were collected at and near the terminus of two perennial streams in northwest Idaho, where the Lapwai and Lawyer Creeks discharge into the Clearwater River, creating delta deposits at the convergence of these creeks and rivers in the towns of Nez Perce (NP004) and Kamiah (KAM001 and 003), respectively (*Thorpe and Hurowitz*, 2020). CIA values calculated only for the mud sized sediments (<63 microns). |
| *Thorpe et al*., (2019) | Fluvial basaltic sediments from Iceland. Geological age assumed from the oldest age of the period identified in the citation. Starting in the upper reaches of the watershed, *Thorpe et al*., (2019) collected a sample of the Icelandic basaltic provenance, and then collected unconsolidated sediment samples along the Hvítá S and its tributaries in the towns of Sandvatan, Laugarás, and Ölfus (samples SV10A, LG002, and OS001, listed with distance from the source progressively increasing). CIA values calculated only for the mud sized sediments (<63 microns). |
| *DeCarlo et al*., (2005) | Streambed sediments from O'ahu Hawai'i. MAP values were determined from the general coordinates for field locations on *Decarlo et al*., 2005 and the Geography Department’s climate database from University of Hawaii. Please see: http://rainfall.geography.hawaii.edu/interactivemap.html. and the following references:  ^1^*Giambelluca, T.W., X. Shuai, M.L. Barnes, R.J. Alliss, R.J. Longman, T. Miura, Q. Chen, A.G. Frazier, R.G. Mudd, L. Cuo, and A.D. Businger*. 2014. Evapotranspiration of Hawai‘i. Final report submitted to the U.S. Army Corps of Engineers—Honolulu District, and the Commission on Water Resource Management, State of Hawai‘i.  ^2^*Frazier, A. G., Giambelluca, T. W., Diaz, H. F. and Needham, H. L*. (2016), Comparison of geostatistical approaches to spatially interpolate month-year rainfall for the Hawaiian Islands. *Int. J. Climatol.*, 36(3), 1459-1470. doi: 10.1002/joc.4437  ^3^*Frazier, A. G., and Giambelluca, T. W*. (2017), Spatial trend analysis of Hawaiian rainfall from 1920 to 2012. *Int. J. Climatol.*, 37(5), 2522–2531. doi: 10.1002/joc.4862 |
| *Craig and Loughnan*, (1964) | Chemical and mineralogical transformations accompanying the weathering of basic volcanic rocks from New South Wales. Geological age assumed from the oldest age of the period identified in the citation. The two deepest samples in the profiles were excluded in order to avoid CIA values from pristine source rocks. |
| *Singh*, (2009) | *This is the only non-mafic sourced reference, as the provenance of these fluvial sediments is from the Himalayan Crystalline Series (HHCS), Lesser Himalayas Series (LHS) and the Siwalik foreland basin. However, this is a detailed study that explores sediments across a stream channel. This data is only compiled in supplementary table s4 and in supplementary Figure s8 and discussed briefly in the text as a potential variable influencing CIA. |

**Text s2:** *Discussion of CaO**

While the CIA value (%) is generally corrected for the calcium that resides only in the silicate fraction of sediments or sedimentary rocks, denoted in the literature as CaO* (*Nesbitt and Young*, 1982; *Nesbitt and Wilson*, 1992; *McLennan et al*., 1993), we chose to ignore this correction and instead use the as-measured CaO abundances, largely because the typically employed CaO* calculation does not account for the thermodynamic and kinetic behavior of Ca-bearing pyroxenes, which are an important component of mafic igneous rocks. We also note that for the martian targets, using the uncorrected CIA value is preferable because APXS only measures the bulk chemical geochemistry, preventing an understanding of whether a significant component of the Ca resides in non-silicate phases (*Siebach and McLennan*, 2018). Therefore, this uncorrected CIA value is a minimum and any sort of correction would only increase the CIA values (*Siebach and McLennan*, 2018). We note that *Curiosity* already has the ability to test this hypothesis of a CIA minimum with the implementation of two geochemical datasets with varying spot sizes, i.e., data from the APXS and CheCam instruments. For example, when examining the CheCam data from the Murray formation, *Mangold et al*., (2019) avoided calcium sulfate veins and averaged multiple points per target for a bulk estimate. In doing, *Mangold et al*., (2019) identified CIA values that exceed the APXS derived CIA values, further demonstrating that the CIA values in this work should be regarded as an alteration baseline.

**Text s3:** *Weighted Average of Terrestrial Muds and Hypothetical Mudrock*

While the CIA values for both terrestrial analogs reach maximum values that are higher than those observed in the Sheepbed and Murray HP targets, we note that the highest terrestrial CIA values are found in the clay size fraction (<2 μm). Direct comparison between the CIA values of clay sized particles and mudstones *sensu stricto* is complicated by the fact that mudstones are composed of a mixture of grain sizes, including some particles >2 μm. Mud and mudrock are generic terms that include silt or siltstones (< 1/3 clay-sized particles), mud or mudstones (subequal parts silt and clay), and clay or claystones (> 2/3 clay) (*Folk*, 1974). On Mars, we are limited in our resolution to determine grain sizes <30 μm (*Siebach et al*., 2017), and therefore unable to determine the relative proportions of clay and silt particles. In order to assess the impact of the proportion of clay particles on a hypothetical terrestrial mudrock bulk composition, we performed a modeling exercise by varying the ratio of silt (<63 μm) to clay (<2 μm) of terrestrial sediments from Iceland (*Thorpe et al.*, 2019) and Idaho (*Thorpe and Hurowitz*, 2020). For this modeling exercise, weighted CIA value averages were calculated for a clay to silt ratio extending from 0 to 1, with results presented in supplementary Table s4 and Figure 3. In this model the systematic mixing of silt and clay compositions from the Iceland and Idaho reference sediments creates a linear mixing curve between the two endmembers, thus documenting a hypothetical mudrock composition extending from claystone to siltstone (Fig. 3). The actual yield of clay sized sediments from the separation technique used in *Thorpe et al*., 2019 and *Thorpe and Hurowitz* 2020 varies between study sites and depositional sites, but the <2 μm sediment accounts for approximately 5 wt% on average and is a good first approximation for the bulk CIA values. This is interesting because a mudrock formed from the terrestrial fluvial sediments in Iceland and Idaho would fall in the siltstone category. Results from this model indicate that grain size likely exerts an additional important control on the observed CIA values of martian mudstones.

**Text s4**: *Weathering Profiles vs. Fluvial Sediments*

In addition to changes in the influence of temperature on CIA values, we observe a fundamental difference between weathering profiles and fluvial sediments, with fluvial sediments demonstrating an offset to lower CIA values under the same climate conditions. This observation suggests a variable extent of chemical weathering between weathering profiles and fluvial sediments, likely controlled by the influence of physical erosion. That is to say, in a weathering profile, where chemical weathering exceeds erosion, the leaching of cations is greater across the whole range of temperature and precipitation values. The opposite is true for fluvial sediments, where physical erosion and transport also play an important role, delivering “fresher” detritus that likely has insufficient time to chemically weather in the fluvial environment as compared to material in weathering profiles. This observation may be important when interpreting if sedimentary rocks in a basin experience alteration in the source terrains and/or along the transportation pathway.

**Supplementary Figures**


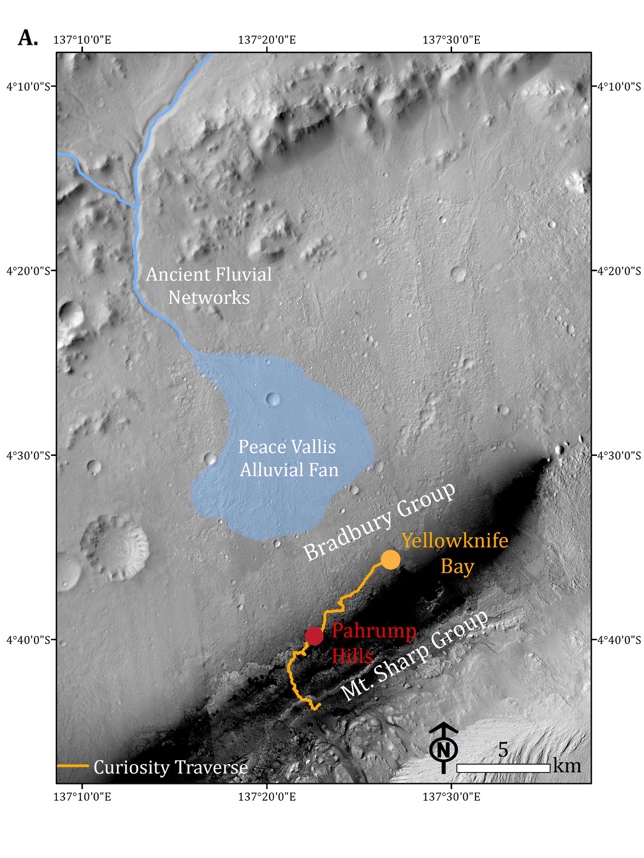

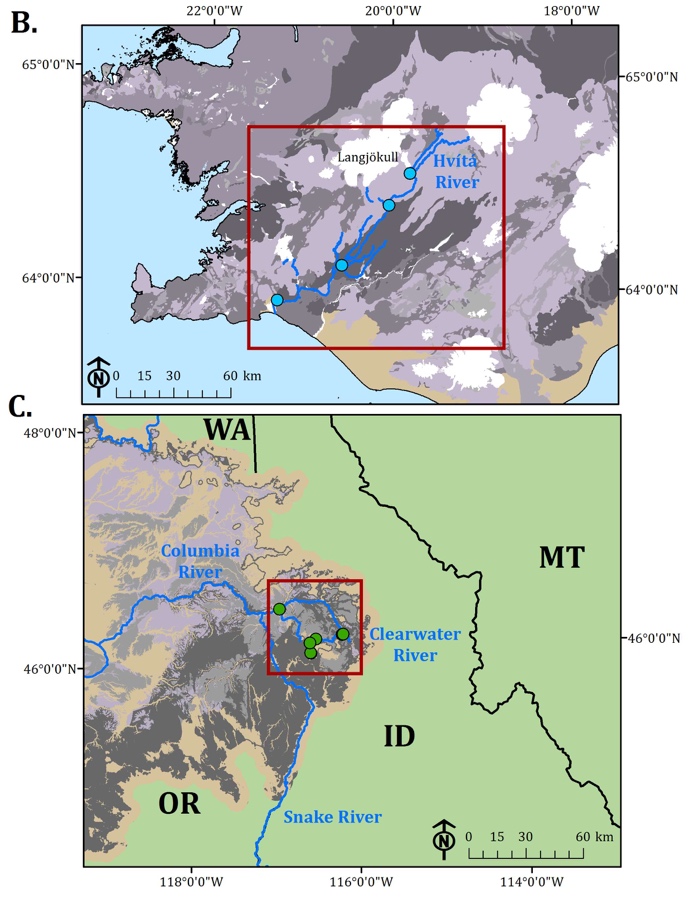


**Supplementary Figure S1**. Martian and terrestrial study sites. In panel (a), a digital elevation model (DEM) created by the U.S. Geological Survey using stereo pairs from the High-Resolution Imaging Science Experiment (HiRISE) (*Parker and Calef*, 2016) is overlaid with the MSL Curiosity rover traverse (orange). Additionally, general target localities for Sheepbed (orange circle) and Murray (red circle) mudstone targets are identified. We also show geomorphological features (overlain in blue) that are interpreted as an ancient fluvial network terminating into an alluvial fan (*Palucis et al*., 2014). For the Earth-based field sites, Iceland (b) and Idaho (c), the spatial extent of basaltic rocks is displayed in various shades of grey. Specific field sites for source-to-sink analysis are designated by red squares and individual sample locations within each site are identified with blue and green circles for Iceland and Idaho, respectively.


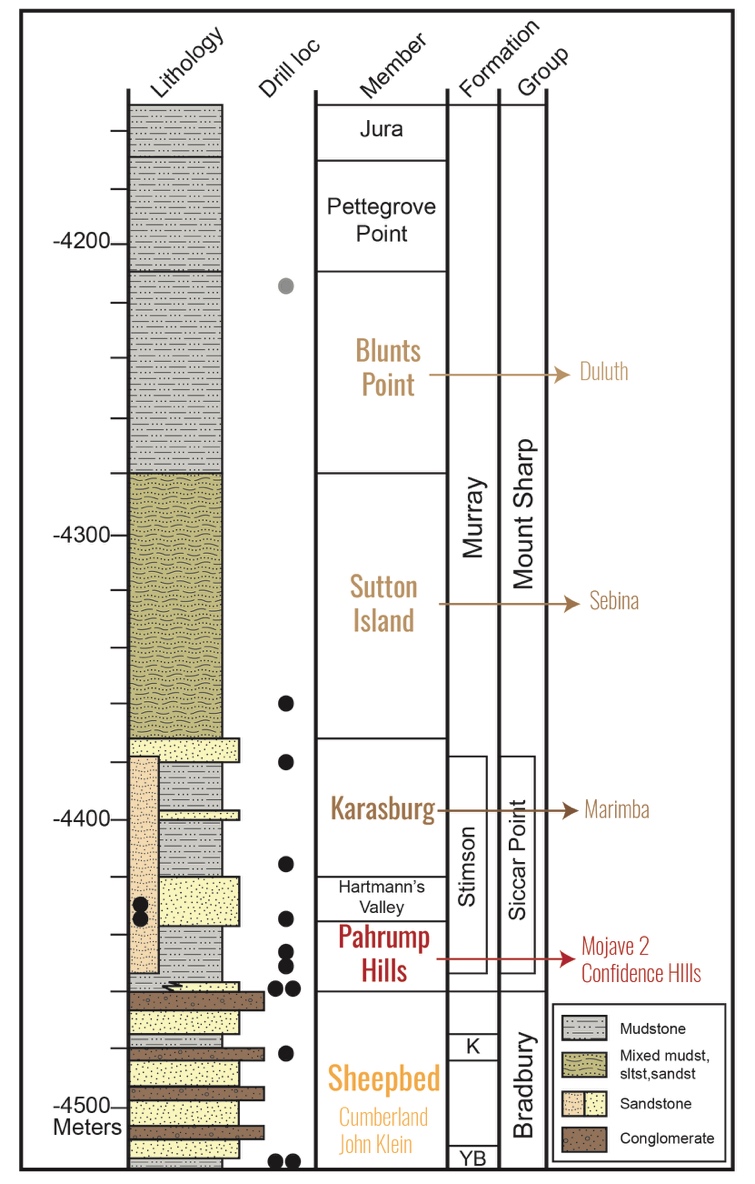


**Supplementary Figure S2**. A stratigraphic column of Bradbury and Mount Sharp group, with the general location of APXS and CheMin samples identified throughout. This figure was reproduced from *Fedo et al*., (2019) and *Edgar* (2020), and updated to display the members used for geochemistry in color and the names of drill targets from Curiosity used for mineralogy throughout this work.


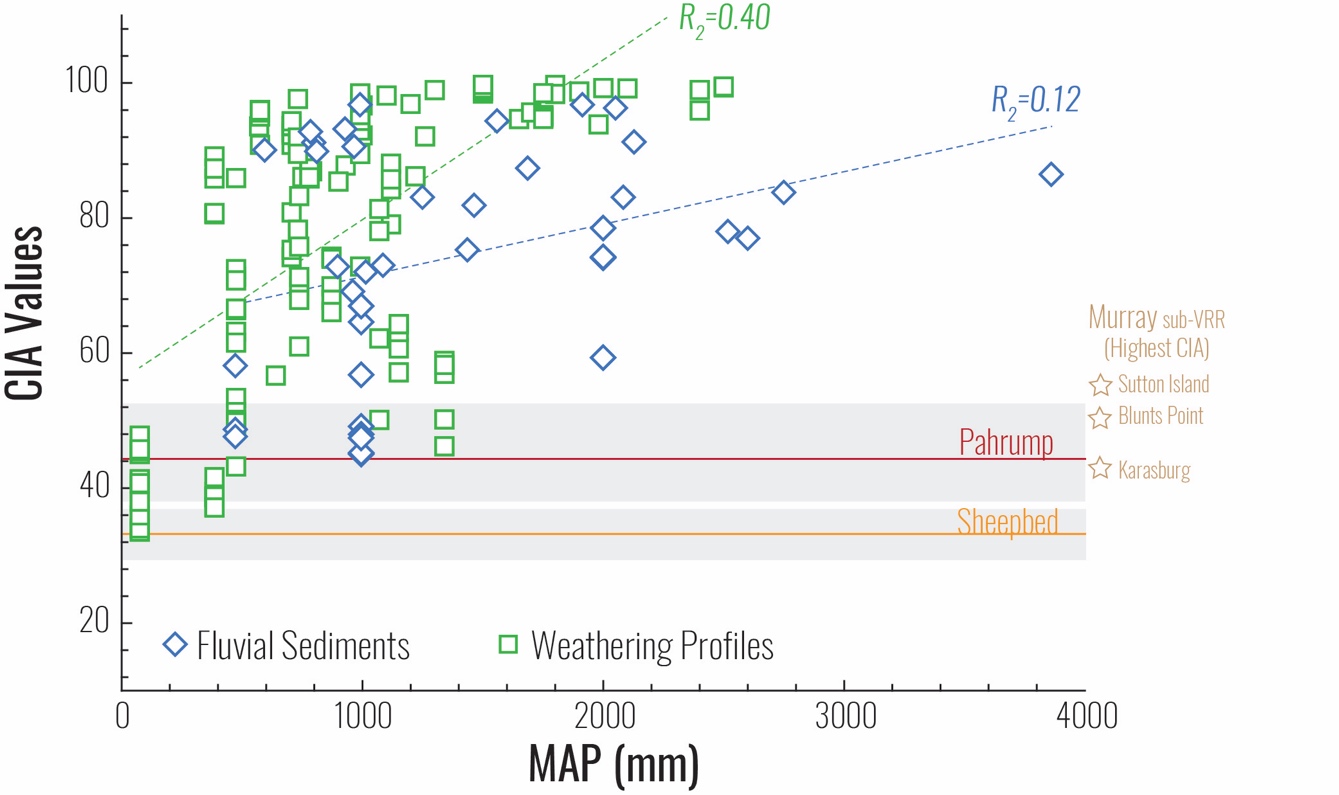


**Supplementary Figure S3**. Chemical index of alteration (CIA) values plotted against mean annual precipitation (MAP) compiled from the literature in comparison to CIA ranges (gray box) and averages (solid lines) Sheepbed and Pahrump mudstones, while mudstones from higher in the Murray stratigraphy are labeled with the highest CIA value plotted as a point (star) on the right side of the diagram and can represent the full range MAP values.


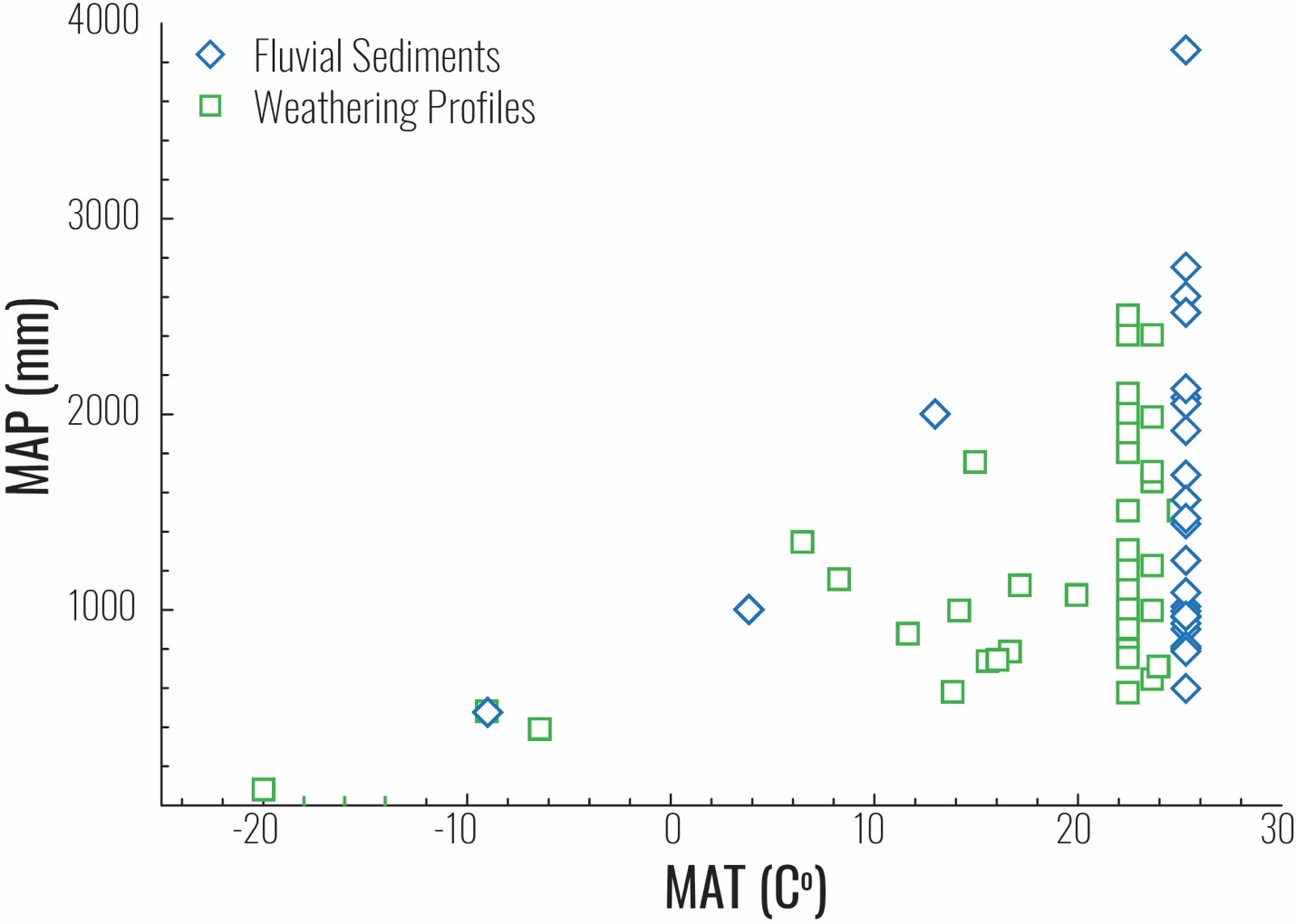


**Supplementary Figure S4**. Mean annual precipitation (MAP) plotted against mean annual temperature (MAT).


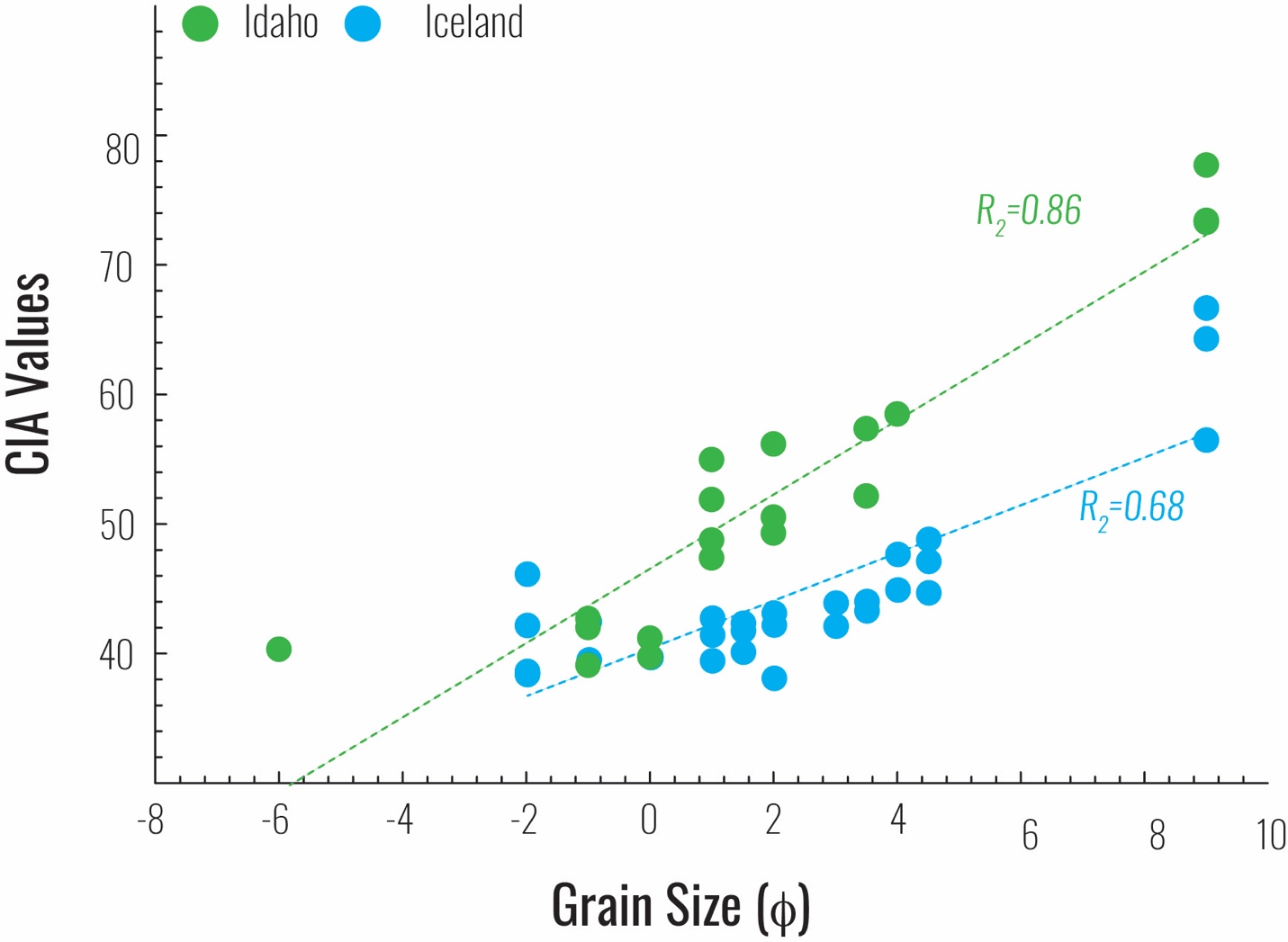


**Supplementary Figure S5**. Chemical Index of Alteration (CIA) values plotted again grain size.

References:

*Thorpe et al*., (2019)

*Thorpe and Hurowitz*, (2020)


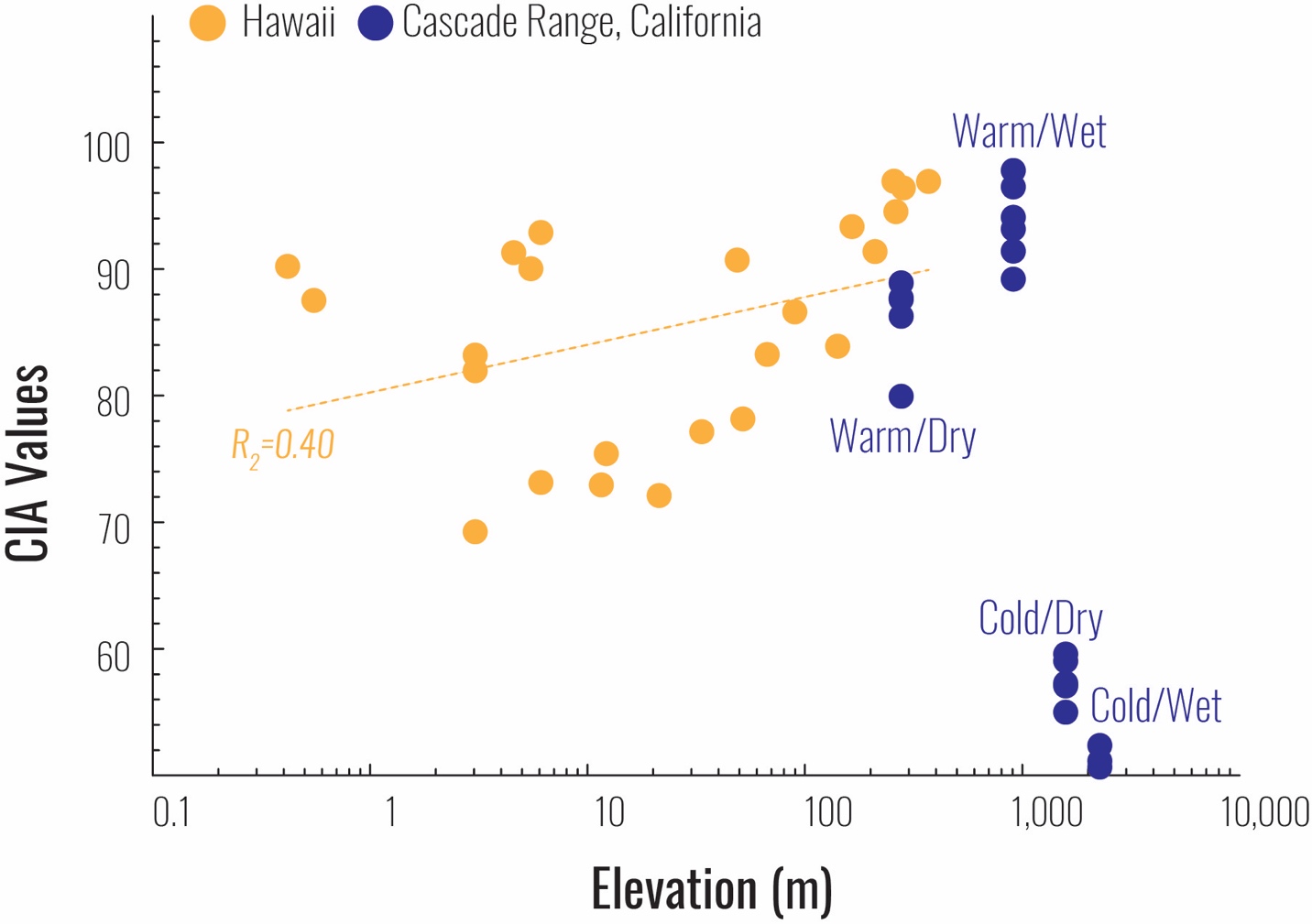


**Supplementary Figure S6**. Chemical Index of Alteration (CIA) values plotted against elevation.

References:

*De Carlo et al*., (2005)

*Rasmussen et al*., (2010)


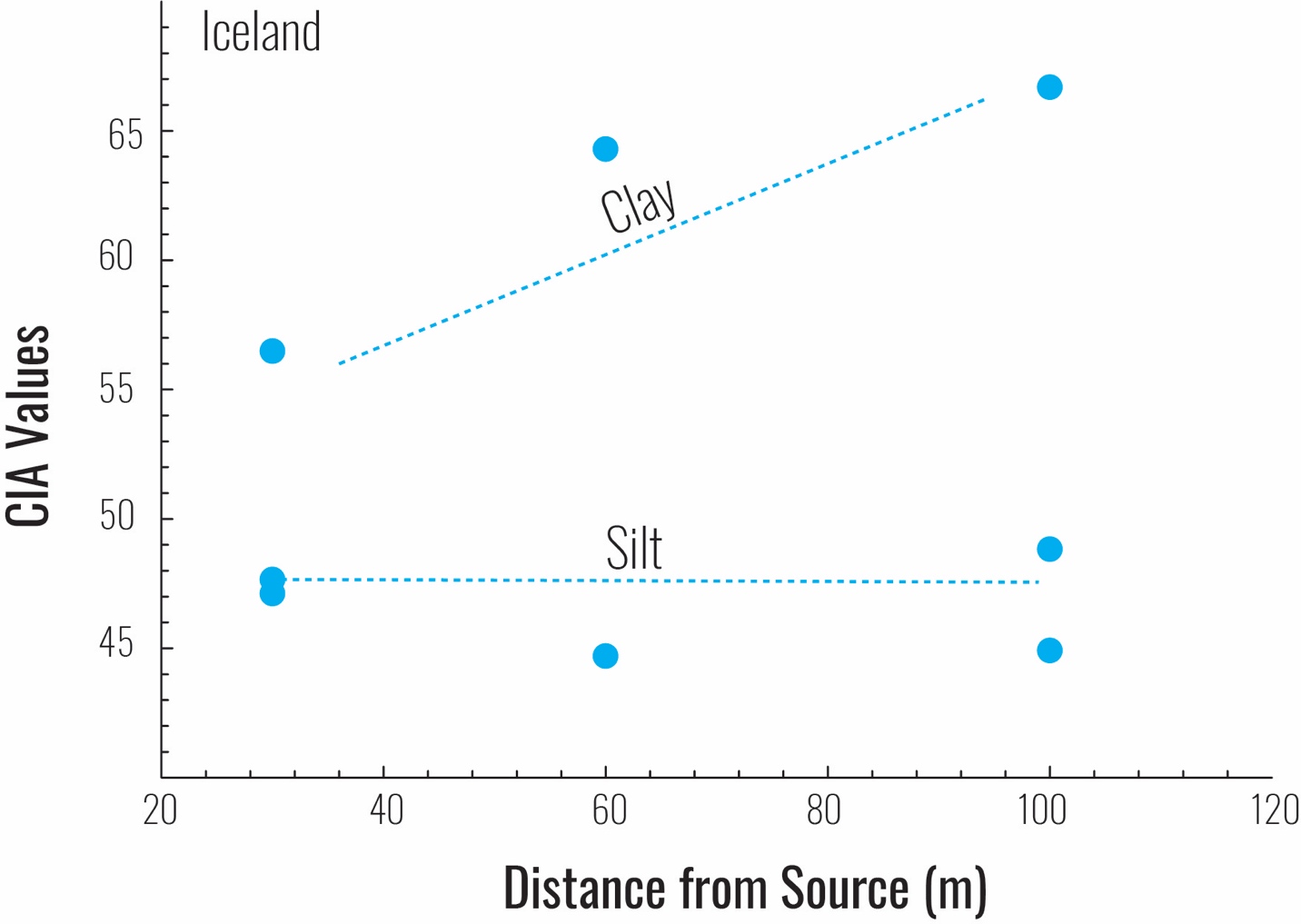


**Supplementary Figure S7**. CIA vs. distance from the source.

Reference:

*Thorpe et al.,* (2019)


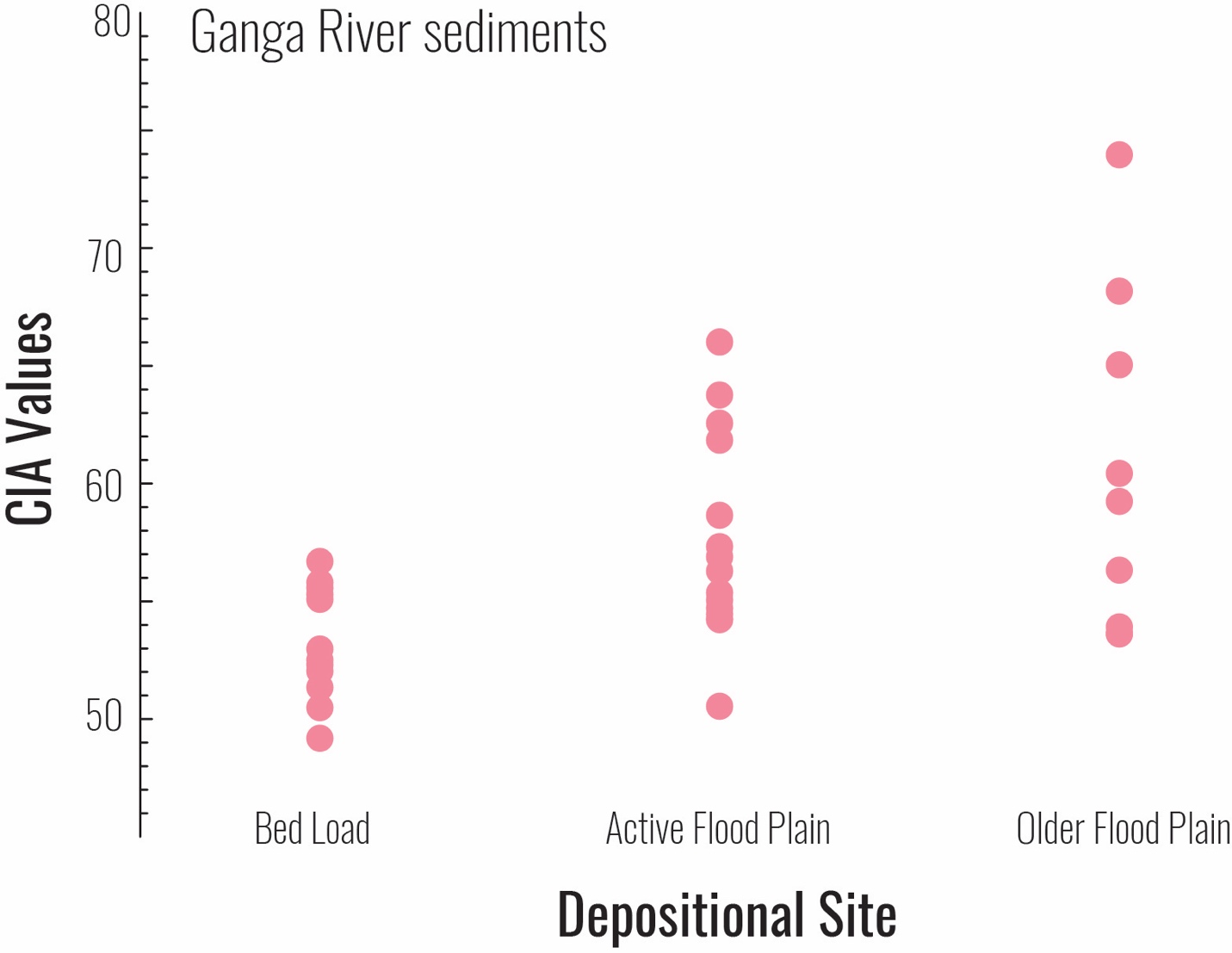


**Supplementary Figure S8**. Chemical Index of Alteration (CIA) values versus depositional site.

Reference:

*Singh*, (2009)


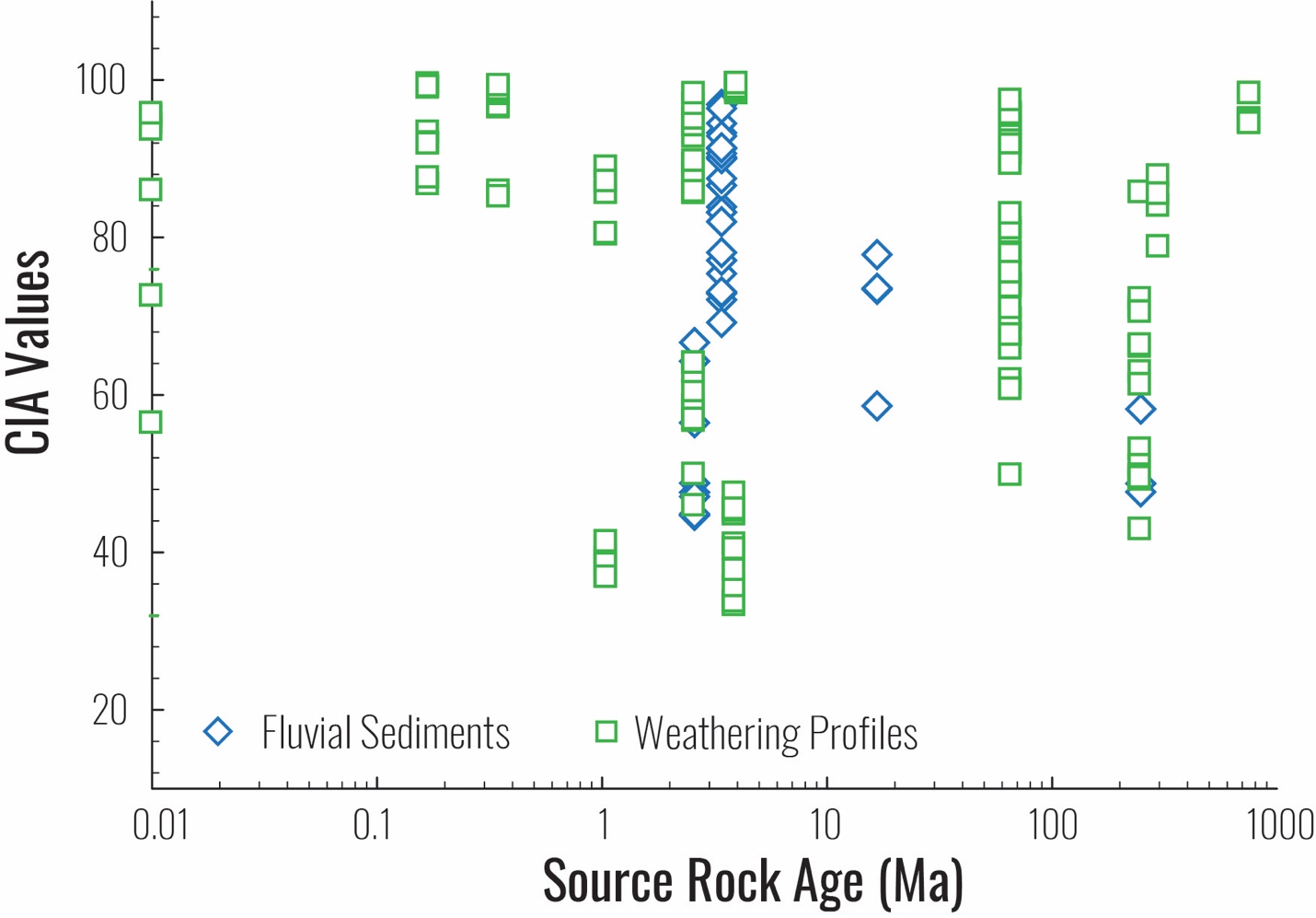


**Supplementary Figure S9**. Chemical Index of Alteration (CIA) values versus source rock age.

References: See Text s1 *(Additional References and Descriptions)*

|  |  |  |  |  |  |  |  |  |  |  |  |  |  |
| --- | --- | --- | --- | --- | --- | --- | --- | --- | --- | --- | --- | --- | --- |
|  | Solar Day (sol) | Target | SiO2 | TiO2 | Al2O3 | FeOT | MnO | MgO | CaO | Na2O | K2O | P2O5 | CIA |
| **Martian Crust^1^** | - | - | 49.48 | 0.98 | 10.54 | 18.27 | 0.36 | 9.09 | 6.95 | 2.98 | 0.45 | 0.90 | 36.90 |
| **Sheepbed Mudstones^2^** | 129 | Costello | 46.13 | 1.13 | 9.11 | 23.04 | 0.44 | 9.51 | 7.18 | 2.39 | 0.50 | 0.56 | 34.20 |
|  | 129 | Flaherty | 45.10 | 1.04 | 8.94 | 23.55 | 0.40 | 9.45 | 7.23 | 2.82 | 0.46 | 1.01 | 32.84 |
|  | 149 | Ekwir_1 | 47.35 | 1.01 | 8.80 | 22.09 | 0.28 | 9.17 | 6.80 | 2.77 | 0.60 | 1.13 | 33.39 |
|  | 150 | Grit_RP | 46.22 | 1.10 | 9.06 | 22.47 | 0.35 | 9.47 | 6.95 | 2.69 | 0.54 | 1.14 | 33.93 |
|  | 150 | Ekwir_1_postbrush | 47.67 | 0.93 | 8.72 | 21.36 | 0.23 | 10.17 | 6.21 | 2.93 | 0.65 | 1.13 | 34.14 |
|  | 154 | Persillon | 45.71 | 0.97 | 8.94 | 21.60 | 0.26 | 9.22 | 8.77 | 2.80 | 0.60 | 1.12 | 29.67 |
|  | 158 | Nastapoka | 47.73 | 1.01 | 9.38 | 21.80 | 0.23 | 9.45 | 5.73 | 2.83 | 0.65 | 1.20 | 37.29 |
|  | 161 | Bonnet_Plume_2_and_APXS_RP | 48.07 | 0.86 | 9.12 | 21.60 | 0.27 | 9.56 | 6.28 | 2.61 | 0.61 | 1.01 | 35.79 |
|  | 161 | Hudson_Bay_dust_free | 47.74 | 1.09 | 9.02 | 22.02 | 0.36 | 9.32 | 6.45 | 2.63 | 0.59 | 0.78 | 35.07 |
|  | 161 | Yukon | 47.91 | 1.10 | 10.86 | 18.75 | 0.39 | 8.42 | 7.58 | 3.22 | 0.70 | 1.06 | 35.37 |
|  | 162 | Hay_Creek_marsviewer_tweaked | 46.55 | 1.03 | 8.93 | 22.65 | 0.35 | 9.53 | 6.36 | 2.90 | 0.57 | 1.14 | 34.51 |
|  | 165 | Sayunei_RP_c | 47.34 | 0.99 | 10.19 | 20.16 | 0.39 | 9.43 | 6.42 | 3.11 | 0.87 | 1.09 | 36.49 |
|  | 165 | Sayunei_RP_d | 45.82 | 0.93 | 8.52 | 23.76 | 0.39 | 10.68 | 5.70 | 2.70 | 0.47 | 1.03 | 35.72 |
|  | 168 | John Klein | 45.78 | 1.17 | 9.09 | 22.48 | 0.34 | 9.21 | 7.63 | 2.74 | 0.64 | 0.92 | 32.29 |
|  | 168 | Wernecke | 46.85 | 1.03 | 9.10 | 22.27 | 0.31 | 9.31 | 6.53 | 2.88 | 0.61 | 1.10 | 34.50 |
|  | 169 | Brock_Inlier_RP | 47.26 | 1.03 | 9.10 | 21.87 | 0.33 | 9.49 | 6.52 | 2.69 | 0.57 | 1.13 | 35.00 |
|  | 169 | Wernecke_Brushed | 48.16 | 0.93 | 9.12 | 21.06 | 0.30 | 10.07 | 5.55 | 3.12 | 0.64 | 1.05 | 36.44 |
|  | 173 | Wernecke_3_Brushed | 48.98 | 0.94 | 9.16 | 20.28 | 0.28 | 10.05 | 5.48 | 3.07 | 0.71 | 1.05 | 36.74 |
|  | 181 | Percuss_Checkout_Divot2 | 46.07 | 1.01 | 9.18 | 21.70 | 0.32 | 10.43 | 6.52 | 3.14 | 0.62 | 1.00 | 34.17 |
|  | 230 | APXS_Drill_Site_Raster_Integration_Site1 | 44.52 | 1.04 | 9.60 | 20.62 | 0.23 | 10.06 | 8.48 | 3.76 | 0.64 | 1.04 | 30.10 |
|  | 230 | APXS_Drill_Site_Raster_Integration_Site2 | 46.26 | 1.10 | 9.10 | 21.12 | 0.32 | 9.59 | 7.82 | 3.10 | 0.61 | 0.97 | 31.30 |
|  | 230 | APXS_Drill_Site_Raster_Integration_Site3 | 45.32 | 1.15 | 8.77 | 21.57 | 0.30 | 9.74 | 8.64 | 2.75 | 0.58 | 1.18 | 29.58 |
|  | 230 | APXS_Drill_Site_Raster_Integration_Site4 | 45.09 | 1.06 | 9.18 | 21.55 | 0.33 | 9.68 | 8.28 | 3.21 | 0.64 | 0.96 | 30.38 |
|  | 230 | APXS_Drill_Site_Raster_Integration_Site5 | 45.19 | 1.02 | 9.44 | 21.07 | 0.27 | 9.77 | 8.37 | 3.24 | 0.58 | 1.04 | 30.84 |
|  | 230 | APXS_Drill_Site_Raster_Integration_Site6 | 44.41 | 1.03 | 9.14 | 21.59 | 0.28 | 9.95 | 8.59 | 3.36 | 0.59 | 1.06 | 29.55 |
|  | 230 | APXS_Drill_Site_Raster_Integration_Site7 | 44.21 | 1.13 | 9.16 | 22.27 | 0.35 | 9.66 | 8.47 | 3.16 | 0.59 | 0.99 | 30.14 |
|  | 270 | McGrath_1 | 47.09 | 1.09 | 9.28 | 22.14 | 0.32 | 9.36 | 6.49 | 2.74 | 0.67 | 0.82 | 35.29 |
|  | 270 | McGrath_2 | 47.02 | 0.88 | 7.18 | 25.44 | 0.40 | 11.12 | 4.72 | 2.16 | 0.32 | 0.74 | 36.52 |
|  | 270 | McGrath_3 | 47.13 | 0.99 | 9.33 | 21.30 | 0.33 | 9.33 | 7.25 | 2.84 | 0.55 | 0.95 | 33.61 |
|  | 270 | McGrath_4 | 46.53 | 1.05 | 8.93 | 22.02 | 0.31 | 9.73 | 7.05 | 2.84 | 0.48 | 1.07 | 33.14 |
|  | 270 | McGrath_center | 46.21 | 0.94 | 7.86 | 24.58 | 0.38 | 10.76 | 5.43 | 2.50 | 0.39 | 0.93 | 35.31 |
|  | 276 | CumberlandNew_RP | 47.39 | 1.05 | 9.20 | 21.93 | 0.28 | 9.36 | 6.33 | 2.79 | 0.56 | 1.10 | 35.50 |
|  | 277 | CumberlandNew_RP_postLIBS | 47.65 | 1.01 | 9.17 | 21.84 | 0.31 | 9.35 | 6.35 | 2.76 | 0.52 | 1.03 | 35.53 |
|  | 283 | Cumberland_A_adjusted_Target1 | 45.96 | 0.93 | 8.81 | 23.71 | 0.25 | 9.74 | 6.58 | 2.70 | 0.49 | 0.83 | 34.23 |
|  | 283 | Cumberland_A_adjusted_Target2 | 46.53 | 1.04 | 9.13 | 22.30 | 0.31 | 9.60 | 6.54 | 3.14 | 0.57 | 0.83 | 34.05 |
|  | 287 | Cumberland_A_adjusted_Target1 | 44.99 | 1.03 | 8.89 | 23.66 | 0.33 | 9.82 | 6.67 | 3.13 | 0.52 | 0.97 | 33.25 |
|  | 291 | Cumberland_DRT_RP_twk_preBrush | 46.85 | 1.11 | 9.45 | 22.19 | 0.38 | 9.06 | 6.87 | 2.65 | 0.61 | 0.84 | 35.06 |
|  | 291 | Cumberland_DRT_RP_twk_postBrush | 48.52 | 0.94 | 9.68 | 20.55 | 0.29 | 9.49 | 5.64 | 3.25 | 0.68 | 0.95 | 37.20 |
|  | 487 | Cumberland_dump_pile_side1 | 45.04 | 0.99 | 9.43 | 22.41 | 0.33 | 9.75 | 7.11 | 3.12 | 0.75 | 1.06 | 33.33 |
|  | 487 | Cumberland_dump_pile_side2 | 44.58 | 1.18 | 9.30 | 22.68 | 0.35 | 10.07 | 6.80 | 3.42 | 0.66 | 0.95 | 33.23 |
|  | 487 | Cumberland_dump_pile_center | 44.01 | 1.06 | 9.23 | 23.49 | 0.31 | 9.97 | 7.13 | 3.22 | 0.66 | 0.92 | 32.74 |
| **Pahrump Mudstones^3^** | 755 | Maturango | 55.34 | 1.14 | 11.13 | 18.06 | 0.33 | 4.80 | 4.02 | 3.11 | 0.92 | 1.15 | 45.31 |
|  | 759 | Confidence_Hills_minidrill | 53.87 | 1.28 | 10.58 | 20.70 | 0.40 | 4.09 | 4.12 | 2.87 | 0.96 | 1.14 | 44.40 |
|  | 762 | Paradox_drillfines | 54.00 | 1.19 | 11.15 | 19.90 | 0.38 | 3.81 | 4.28 | 3.13 | 0.90 | 1.26 | 44.51 |
|  | 767 | Confidence_Hills_fines_presieve_dump | 52.53 | 1.24 | 10.36 | 21.48 | 0.40 | 4.78 | 4.34 | 2.81 | 0.98 | 1.09 | 43.32 |
|  | 781 | Confidence_Hills_fines_postsieve_dump | 51.22 | 1.20 | 10.35 | 21.10 | 0.40 | 5.91 | 4.87 | 2.82 | 1.04 | 1.09 | 41.44 |
|  | 805 | Ricardo_raster1 | 49.00 | 1.21 | 10.19 | 20.94 | 0.58 | 8.06 | 5.45 | 2.69 | 0.85 | 1.02 | 40.07 |
|  | 805 | Ricardo_raster2 | 52.04 | 1.26 | 10.92 | 17.19 | 0.36 | 7.88 | 5.53 | 2.89 | 0.86 | 1.07 | 40.97 |
|  | 805 | Pelona_DRT | 54.95 | 1.18 | 11.23 | 17.66 | 0.36 | 5.39 | 4.28 | 2.95 | 0.91 | 1.09 | 45.21 |
|  | 806 | Ricardo_raster1_DRT | 54.07 | 1.17 | 10.89 | 18.74 | 0.54 | 5.18 | 3.98 | 2.97 | 1.03 | 1.44 | 45.13 |
|  | 809 | Mojave_DRT_raster1 | 55.09 | 1.13 | 13.44 | 15.09 | 0.37 | 5.15 | 4.62 | 2.91 | 0.74 | 1.46 | 48.98 |
|  | 809 | Mojave_DRT_raster2 | 55.83 | 1.15 | 13.36 | 14.55 | 0.37 | 4.81 | 4.62 | 3.06 | 0.70 | 1.55 | 48.50 |
|  | 813 | Punchbowl_DRT_raster1 | 56.96 | 1.09 | 13.17 | 13.09 | 0.33 | 6.06 | 4.27 | 2.89 | 0.81 | 1.33 | 49.58 |
|  | 813 | Punchbowl_DRT_raster2 | 56.23 | 1.13 | 12.93 | 14.13 | 0.34 | 5.75 | 4.35 | 2.93 | 0.81 | 1.42 | 48.73 |
|  | 814 | Afton_Canyon_DRT_raster1 | 55.39 | 1.18 | 13.37 | 15.07 | 0.32 | 5.80 | 4.03 | 2.93 | 0.80 | 1.10 | 50.67 |
|  | 814 | Afton_Canyon_DRT_raster2 | 56.16 | 1.13 | 14.01 | 14.27 | 0.29 | 5.39 | 3.79 | 2.91 | 0.86 | 1.19 | 52.61 |
|  | 815 | Topanga_DRT_raster1 | 55.58 | 1.17 | 12.32 | 15.18 | 0.33 | 6.04 | 4.45 | 2.96 | 0.79 | 1.17 | 47.14 |
|  | 815 | Topanga_DRT_raster2 | 58.45 | 1.15 | 13.32 | 12.56 | 0.30 | 5.25 | 3.95 | 2.99 | 0.84 | 1.20 | 50.62 |
|  | 819 | Mescal_DRT_raster1 | 56.27 | 1.03 | 12.14 | 16.07 | 0.34 | 4.66 | 4.44 | 2.99 | 0.81 | 1.25 | 46.66 |
|  | 820 | Mescal_DRT_raster2 | 55.29 | 1.02 | 11.89 | 16.15 | 0.34 | 5.68 | 4.69 | 2.93 | 0.78 | 1.23 | 45.58 |
|  | 824 | Puente_DRT_raster1 | 56.35 | 1.06 | 10.96 | 16.44 | 0.30 | 5.64 | 4.57 | 2.69 | 0.81 | 1.18 | 44.61 |
|  | 824 | Puente_DRT_raster2 | 56.08 | 1.03 | 11.39 | 16.74 | 0.32 | 5.21 | 4.33 | 2.89 | 0.80 | 1.22 | 45.77 |
|  | 828 | Pickhandle_DRT | 55.54 | 1.17 | 11.92 | 16.49 | 0.29 | 5.49 | 4.29 | 2.92 | 0.80 | 1.08 | 46.94 |
|  | 830 | Goldstone_DRT_raster1 | 54.95 | 1.07 | 11.40 | 16.09 | 0.25 | 6.27 | 5.53 | 2.71 | 0.79 | 0.93 | 42.59 |
|  | 831 | Goldstone_DRT_raster2 | 53.04 | 1.07 | 10.93 | 16.45 | 0.26 | 6.89 | 6.83 | 2.74 | 0.75 | 1.04 | 38.13 |
|  | 833 | Tropico | 52.80 | 1.12 | 10.53 | 17.21 | 0.29 | 7.92 | 5.50 | 2.82 | 0.76 | 1.04 | 40.49 |
|  | 867 | Mojave_mini_drill | 52.85 | 1.24 | 13.72 | 16.84 | 0.38 | 4.80 | 5.04 | 2.75 | 0.81 | 1.58 | 48.52 |
|  | 871 | Mojave_chunk | 54.60 | 1.16 | 14.04 | 14.99 | 0.36 | 4.92 | 4.55 | 3.02 | 0.72 | 1.64 | 50.03 |
|  | 880 | Mojave2 | 55.73 | 1.18 | 12.70 | 14.47 | 0.39 | 5.43 | 4.75 | 3.00 | 0.76 | 1.59 | 46.86 |
|  | 881 | Mojave2_mini_drill | 54.30 | 1.29 | 13.22 | 16.17 | 0.41 | 4.15 | 4.79 | 3.28 | 0.79 | 1.59 | 46.90 |
|  | 884 | Mojave2_DTc_drilltailings | 54.93 | 1.26 | 12.72 | 15.63 | 0.43 | 4.79 | 4.84 | 3.20 | 0.67 | 1.52 | 46.23 |
|  | 888 | Mojave2_presieve_dump | 53.96 | 1.19 | 12.42 | 16.73 | 0.41 | 5.07 | 4.81 | 3.24 | 0.79 | 1.38 | 45.40 |
|  | 889 | San_Francisquito_raster1 | 53.28 | 1.13 | 11.75 | 16.60 | 0.37 | 6.48 | 5.49 | 2.67 | 0.72 | 1.51 | 43.67 |
|  | 894 | Mojave2_postsieve_dump | 53.48 | 1.29 | 12.35 | 17.41 | 0.43 | 4.92 | 4.68 | 3.25 | 0.79 | 1.39 | 45.64 |
|  | 1157 | Augusta_DRT_offset | 54.85 | 1.10 | 10.02 | 18.85 | 0.24 | 5.34 | 5.20 | 2.76 | 1.06 | 0.57 | 39.81 |
|  | 1157 | Augusta_DRT_centre | 56.23 | 1.10 | 9.90 | 18.53 | 0.24 | 4.88 | 4.77 | 2.71 | 1.13 | 0.51 | 40.81 |
|  | 1166 | Swartkloofberg_DRT | 55.02 | 1.14 | 10.00 | 18.44 | 0.35 | 6.03 | 4.32 | 2.91 | 1.00 | 0.79 | 42.16 |
|  | 1191 | Elizabeth_Bay | 50.89 | 1.13 | 10.39 | 17.89 | 0.30 | 8.45 | 6.12 | 2.90 | 0.66 | 1.25 | 38.46 |
|  | 1259 | Groot_Aub | 52.13 | 1.05 | 9.47 | 19.55 | 0.30 | 7.58 | 4.96 | 2.79 | 1.06 | 1.10 | 39.09 |
|  | 1259 | Gorob_DRT | 53.27 | 1.09 | 9.96 | 19.03 | 0.30 | 6.67 | 4.97 | 2.87 | 0.93 | 0.91 | 40.28 |
|  | 1266 | Waterberg | 53.70 | 1.07 | 9.63 | 19.54 | 0.28 | 6.34 | 4.64 | 2.75 | 1.15 | 0.89 | 40.37 |
|  | 1266 | Stockdale_DRT | 55.41 | 1.11 | 10.03 | 18.15 | 0.28 | 5.50 | 4.73 | 2.78 | 1.31 | 0.71 | 40.77 |
|  | 1273 | Kleinberg | 52.24 | 1.01 | 10.44 | 19.39 | 0.17 | 6.13 | 5.60 | 2.75 | 1.11 | 1.17 | 39.62 |
|  | 1273 | Schwarzrand_DRT | 53.78 | 1.11 | 11.01 | 17.56 | 0.13 | 6.13 | 5.14 | 2.85 | 1.19 | 1.09 | 41.79 |
|  | 1275 | Mirabib_DRT | 55.09 | 1.11 | 10.12 | 19.18 | 0.32 | 5.31 | 4.16 | 2.69 | 1.12 | 0.90 | 43.40 |
| **Karasburg^4^** | 1444 | Ganda_DRT | 52.01 | 1.17 | 9.37 | 22.60 | 0.16 | 6.12 | 4.20 | 2.33 | 0.85 | 1.20 | 43.05 |
| **Sutton Island^4^** | 1531 | Precipice_DRT | 55.46 | 1.15 | 10.31 | 22.95 | 0.08 | 3.58 | 2.16 | 2.26 | 0.86 | 1.19 | 54.60 |
| **Blunts Point^4^** | 1736 | Winter_Harbor | 52.37 | 1.23 | 9.27 | 24.26 | 0.32 | 5.46 | 2.72 | 2.11 | 1.06 | 1.20 | 49.19 |

**Table s1**. Mars Geochemistry

References:

^1^*Hahn and McLennan*, 2007; *Taylor and McLennan*, 2009

^2^*McLennan et al*., (2014)

^3^*Hurowitz et al*., (2017)

^4^*Berger et al*., (2020)

|  |  |  |  |  |  |  |  |  |  |  |  |  |  |  |
| --- | --- | --- | --- | --- | --- | --- | --- | --- | --- | --- | --- | --- | --- | --- |
|  | Gale Crater, Mars | | | |  |  |  | Icelandic Sediment^4^ | | | | | | CRB Sediment^5^ |
|  | Sheepbed^1^ | | Pahrump^2^ | | Karasburg^3^ | Karasburg^3^ | Sutton Island^3^ | SV10A | | LG002 | | OS001 | | NP004 |
| Target or Grain Size (μm) | John Klein | Cumberland | Confidence Hills | Mojave2 | Marimba | Quela | Sebina | <45 | 45-63 | <45 | 45-63 | <45 | 45-63 | <63 |
| Plagioclase | 22.84 | 22.20 | 20.40 | 23.50 | 14.00 | 13.50 | 10.70 | 39.95 | 32.22 | 22.98 | 20.16 | 26.83 | 26.80 | 19.80 |
| Pyroxene | 8.60 | 12.10 | 13.80 | 6.80 | 0.70 | 2.70 | 2.80 | 18.09 | 19.57 | 14.23 | 10.89 | 16.19 | 18.42 | 2.00 |
| Olivine | 2.80 | 0.90 | 1.20 | 0.20 | 0.00 | 0.00 | 0.00 | 3.34 | 4.13 | 1.63 | 1.65 | 2.07 | 3.33 | 0 |
| Clay Minerals | 22.00 | 18.00 | 7.60 | 4.70 | 28.00 | 16.00 | 19.00 | 9.46 | 11.02 | 13.10 | 13.06 | 16.47 | 13.35 | 31.20 |
| X-ray Amorphous | 28.00 | 31.00 | 39.20 | 53.00 | 40.00 | 52.00 | 51.00 | 29.16 | 33.07 | 48.05 | 54.24 | 38.44 | 38.11 | 22.90 |
| K-spar | 1.20 | 1.60 | 5.00 | 0.00 | 2.40 | 2.30 | 1.40 | 0.00 | 0.00 | 0.00 | 0.00 | 0.00 | 0.00 | 7.10 |
| Quartz | 0.4 | 0.1 | 0.7 | 0.8 | 0.5 | 0.5 | 0.5 | 0 | 0 | 0 | 0 | 0 | 0 | 16.50 |

**Table s2**. Martian and Terrestrial Mineralogy

References:

^1^*Vaniman et al*., (2014)

^2^*Rampe et al*., (2017)

^3^*Bristow et al*., (2018)

^4^*Thorpe et al*., (2019)

^5^*Thorpe and Hurowitz* (2020)

|  | Reference | Sample |  | Source Geological Age (Ma) | Mean Annual Temperature (MAT; C^○^) | Mean Annual Precipitation (MAP; mm) | Chemical Index of Alteration (CIA) |
| --- | --- | --- | --- | --- | --- | --- | --- |
| Weathering Profiles | *Rasmussen et al.*, (2010) | Red Fir (RF-4) | A | 2.58 | 6.5 | 1340 | 50.1 |
|  |  | Cold Wet | C |  |  |  | 46.1 |
|  |  |  | 2Ab |  |  |  | 56.8 |
|  |  |  | 2Bwb1 |  |  |  | 58.8 |
|  |  |  | 2Bwb2 |  |  |  | 58.2 |
|  |  | White Fir (WF-3) | A |  | 8.3 | 1150 | 57.1 |
|  |  | Cold Dry | AB |  |  |  | 60.6 |
|  |  |  | Bw1 |  |  |  | 63.0 |
|  |  |  | Bw2 |  |  |  | 64.2 |
|  |  |  | BC |  |  |  | 64.2 |
|  |  | Ponderosa Pine (PP-2) | A |  | 14.2 | 990 | 89.4 |
|  |  | Warm Wet | AB |  |  |  | 93.0 |
|  |  |  | Bt1 |  |  |  | 94.4 |
|  |  |  | Bt2 |  |  |  | 95.2 |
|  |  |  | Bt3 |  |  |  | 97.1 |
|  |  |  | Bt4 |  |  |  | 98.5 |
|  |  | Oak (OK-1) | A |  | 16.7 | 780 | 85.8 |
|  |  | Warm Dry | Bt1 |  |  |  | 88.3 |
|  |  |  | Bt2 |  |  |  | 88.2 |
|  |  |  | Bt3 |  |  |  | 89.9 |
|  |  |  | Bt4 |  |  |  | 86.0 |
|  | *Pokrovsky et al*., (2005) | 23 soil | < 1 μm | 248 | -9 | 475 | 71.2 |
|  |  | 24 soil | < 1 μm |  |  |  | 66.3 |
|  |  | 26 soil | < 1 μm |  |  |  | 66.5 |
|  |  | 28a soil | < 1 μm |  |  |  | 63.1 |
|  |  | 38 soil | < 1 μm |  |  |  | 61.5 |
|  |  | 46 soil | < 1 μm |  |  |  | 72.4 |
|  |  | 47 soil | < 1 μm |  |  |  | 85.9 |
|  |  | 23 soil | 1–50 μm |  |  |  | 53.3 |
|  |  | 24 soil | 1–50 μm |  |  |  | 51.2 |
|  |  | 26 soil | 1–50 μm |  |  |  | 49.4 |
|  |  | 28a soil | 1–50 μm |  |  |  | 50.0 |
|  |  | 38 soil | 1–50 μm |  |  |  | 43.1 |
|  |  | 46 soil | 1–50 μm |  |  |  | 49.7 |
|  |  | 47 soil | 1–50 μm |  |  |  | 70.7 |
|  | *Nesbitt and Wilson* (1992) | A-5 | | 66 | 24 | 705 | 74.2 |
|  |  | A-6 | |  |  |  | 90.8 |
|  |  | A-7 | |  |  |  | 92.7 |
|  |  | A-8 | |  |  |  | 92.3 |
|  |  | A-9 | |  |  |  | 93.6 |
|  |  | C-2 | |  |  |  | 75.2 |
|  |  | C-3 | |  |  |  | 80.8 |
|  |  | C-4 | |  |  |  | 93.6 |
|  |  | C-5 | |  |  |  | 94.3 |
|  | *Ma et al*., (2007) | HK-06 | 1 | 4 | 25 | 1500 | 98.9 |
|  |  | HK-06 | 2 |  |  |  | 98.4 |
|  |  | HK-06 | 3 |  |  |  | 98.8 |
|  |  | HK-06 | 4 |  |  |  | 99.4 |
|  |  | HK-06 | 5 |  |  |  | 99.3 |
|  |  | HK-06 | 6 |  |  |  | 99.5 |
|  |  | HK-06 | 7 |  |  |  | 99.4 |
|  |  | HK-06 | 8 |  |  |  | 99.6 |
|  |  | HK-06 | 9 |  |  |  | 99.6 |
|  |  | HK-06 | 10 |  |  |  | 99.7 |
|  |  | HK-06 | 11 |  |  |  | 99.0 |
|  |  | HK-06 | 12 |  |  |  | 99.7 |
|  |  | HK-06 | 13 |  |  |  | 99.2 |
|  |  | HK-06 | 14 |  |  |  | 99.7 |
|  |  | HK-06 | 15 |  |  |  | 99.7 |
|  |  | HK-06 | 16 |  |  |  | 99.4 |
|  |  | HK-06 | 17 |  |  |  | 99.7 |
|  |  | HK-06 | 18 |  |  |  | 99.2 |
|  |  | HK-06 | 19 |  |  |  | 99.7 |
|  | *Yesavage et al*., (2015) | Sieved (<2mm) | 0–20 cm | 1.05 | -6.4 | 385 | 39.4 |
|  |  |  | 20–38 cm |  |  |  | 41.5 |
|  |  |  | 38–47 cm |  |  |  | 38.6 |
|  |  |  | 47–56 cm |  |  |  | 37.1 |
|  |  |  | 56–63 cm |  |  |  | 38.5 |
|  |  |  | 63–70.5 cm |  |  |  | 38.7 |
|  |  |  | 70.5–74 cm |  |  |  | 37.0 |
|  |  | Clay Fraction (<2 mm) | 0–20 cm |  |  |  | 89.1 |
|  |  |  | 20–38 cm |  |  |  | 85.8 |
|  |  |  | 38–47 cm |  |  |  | 87.3 |
|  |  |  | 47–56 cm |  |  |  | 80.5 |
|  |  |  | 56–63 cm |  |  |  | 80.6 |
|  |  |  | 70.5–74 cm |  |  |  | 80.7 |
|  | *Caner et al*., (2014) | A | 0–25 cm | 760 | 15 | 1750 | 98.5 |
|  |  | AB | 25–45 cm |  |  |  | 95.2 |
|  |  | BA | 45–70 cm |  |  |  | 95.0 |
|  |  | Bw | 70–100 cm |  |  |  | 95.0 |
|  |  | C1 | 100–130 cm |  |  |  | 95.2 |
|  |  | C2 | 100–130 cm |  |  |  | 94.9 |
|  |  | S | 160–190 cm |  |  |  | 94.7 |
|  | *Gibson et al*., (1983) | WV-222 | | 3.9 | -20 | 75 | 41.2 |
|  |  | WV-219 | |  |  |  | 40.6 |
|  |  | WV-238 | |  |  |  | 33.4 |
|  |  | WV-215 | |  |  |  | 45.0 |
|  |  | WV-216 | |  |  |  | 33.9 |
|  |  | WV-217 | |  |  |  | 45.4 |
|  |  | WV-218 | |  |  |  | 35.8 |
|  |  | WV-220 | |  |  |  | 47.7 |
|  |  | WV-214 | |  |  |  | 37.9 |
|  |  | WV-221 | |  |  |  | 45.6 |
|  | *Porder et al*., (2007) | Kona 640mm | | 0.01 | 23.7 | 640 | 56.6 |
|  |  | Kona 990mm | |  | 23.7 | 990 | 72.8 |
|  |  | Kona 1220mm | |  | 23.7 | 1220 | 86.2 |
|  |  | Kona 1650mm | |  | 23.7 | 1650 | 94.6 |
|  |  | Kona 1700mm | |  | 23.7 | 1700 | 95.6 |
|  |  | Kona 1980mm | |  | 23.7 | 1980 | 93.8 |
|  |  | Kona 2400mm | |  | 23.7 | 2400 | 95.9 |
|  |  | Hawi 570mm | | 0.17 | 22.5 | 570 | 93.6 |
|  |  | Hawi 790mm | |  | 22.5 | 790 | 86.9 |
|  |  | Hawi 930mm | |  | 22.5 | 930 | 87.8 |
|  |  | Hawi 1000mm | |  | 22.5 | 1000 | 92.2 |
|  |  | Hawi 1260mm | |  | 22.5 | 1260 | 92.1 |
|  |  | Hawi 1500mm | |  | 22.5 | 1500 | 99.2 |
|  |  | Hawi 1800mm | |  | 22.5 | 1800 | 99.7 |
|  |  | Hawi 2500mm | |  | 22.5 | 2500 | 99.3 |
|  |  | Pololu 750mm | | 0.35 | 22.5 | 750 | 86.1 |
|  |  | Pololu 900mm | |  | 22.5 | 900 | 85.4 |
|  |  | Pololu 1000mm | |  | 22.5 | 1000 | 96.7 |
|  |  | Pololu 1100mm | |  | 22.5 | 1100 | 98.1 |
|  |  | Pololu 1200mm | |  | 22.5 | 1200 | 96.9 |
|  |  | Pololu 1300mm | |  | 22.5 | 1300 | 98.9 |
|  |  | Pololu 1500mm | |  | 22.5 | 1500 | 98.9 |
|  |  | Pololu 1800mm | |  | 22.5 | 1800 | 98.4 |
|  |  | Pololu 1900mm | |  | 22.5 | 1900 | 98.7 |
|  |  | Pololu 2000mm | |  | 22.5 | 2000 | 99.2 |
|  |  | Pololu 2100mm | |  | 22.5 | 2100 | 99.2 |
|  |  | Pololu 2400mm | |  | 22.5 | 2400 | 99.0 |
|  |  | Pololu 2500mm | |  | 22.5 | 2500 | 99.5 |
|  | *Craig and Loughnan*, (1964) | Kiama | K1 | 298.9 | 17.2 | 1117.6 | 84.2 |
|  |  |  | K2 |  | 17.2 | 1117.6 | 88.0 |
|  |  |  | K3 |  | 17.2 | 1117.6 | 85.7 |
|  |  |  | K4 |  | 17.2 | 1117.6 | 79.0 |
|  |  | Bathurst | B1 | 66 | 13.9 | 574.0 | 90.8 |
|  |  |  | B2 |  | 13.9 | 574.0 | 95.0 |
|  |  |  | B3 |  | 13.9 | 574.0 | 95.9 |
|  |  |  | B4 |  | 13.9 | 574.0 | 96.0 |
|  |  |  | B5 |  | 13.9 | 574.0 | 95.6 |
|  |  |  | B6 |  | 13.9 | 574.0 | 95.9 |
|  |  | Inverell | I1 | 66 | 15.6 | 731.5 | 97.6 |
|  |  |  | I2 |  | 15.6 | 731.5 | 91.9 |
|  |  |  | I3 |  | 15.6 | 731.5 | 89.5 |
|  |  |  | I4 |  | 15.6 | 731.5 | 78.2 |
|  |  | Casino | C1 | 66 | 20.0 | 1069.3 | 81.3 |
|  |  |  | C2 |  | 20.0 | 1069.3 | 78.0 |
|  |  |  | C3 |  | 20.0 | 1069.3 | 62.1 |
|  |  |  | C4 |  | 20.0 | 1069.3 | 50.0 |
|  |  | Guyra | G1 | 66 | 11.7 | 871.2 | 69.8 |
|  |  |  | G2 |  | 11.7 | 871.2 | 74.2 |
|  |  |  | G3 |  | 11.7 | 871.2 | 73.9 |
|  |  |  | G4 |  | 11.7 | 871.2 | 67.8 |
|  |  |  | G5 |  | 11.7 | 871.2 | 66.0 |
|  |  | Murrurundi | M1 | 66 | 16.1 | 736.6 | 83.2 |
|  |  |  | M2 |  | 16.1 | 736.6 | 75.7 |
|  |  |  | M3 |  | 16.1 | 736.6 | 69.8 |
|  |  |  | M4 |  | 16.1 | 736.6 | 67.8 |
|  |  |  | M5 |  | 16.1 | 736.6 | 71.2 |
|  |  |  | M6 |  | 16.1 | 736.6 | 60.9 |
| Fluvial Sediments | *Thorpe and Hurowitz*, (2020) | Idaho Source Rock | - | 16.7 | 13 | 2000 | 39.7 |
|  |  | NP004 | <2 μm |  |  |  | 73.4 |
|  |  | NP004 | <63 μm |  |  |  | 58.6 |
|  |  | KAM003 | <2 μm |  |  |  | 73.6 |
|  |  | KAM001 | <2 μm |  |  |  | 77.8 |
|  | *Thorpe et al*., (2019) | Iceland Source Rock | - | 2.58 | 3.85 | 1000 | 37.5 |
|  |  | SV10A | <2 μm |  |  |  | 56.5 |
|  |  | LG002 | <2 μm |  |  |  | 64.3 |
|  |  | OS001 | <2 μm |  |  |  | 66.7 |
|  |  | LG002 | <45 μm |  |  |  | 44.7 |
|  |  | OS001 | <45 μm |  |  |  | 48.8 |
|  |  | OS001 | 45-63 μm |  |  |  | 44.9 |
|  |  | SV10A | 45-63 μm |  |  |  | 47.7 |
|  |  | SV10A | <45 μm |  |  |  | 47.1 |
|  | *DeCarlo et al*., (2005) | <63 μm sediments | U1 | 3.4 | 25.33 | 1016.7 | 72.1 |
|  |  |  | U2 |  |  | 2751.7 | 83.9 |
|  |  |  | U3 |  |  | 1437.9 | 75.4 |
|  |  |  | U4 |  |  | 899.8 | 72.9 |
|  |  |  | U5 |  |  | 961.8 | 69.2 |
|  |  |  | M1 |  |  | 3861.61 | 86.6 |
|  |  |  | M2 |  |  | 1688.4 | 87.5 |
|  |  |  | M3 |  |  | 929.7 | 93.3 |
|  |  |  | M4 |  |  | 992.3 | 96.9 |
|  |  |  | M5 |  |  | 1560.9 | 94.5 |
|  |  |  | M6 |  |  | 800.4 | 91.3 |
|  |  |  | M7 |  |  | 2085.5 | 83.2 |
|  |  |  | M8 |  |  | 1251.8 | 83.2 |
|  |  |  | M9 |  |  | 1087.9 | 73.1 |
|  |  |  | M10 |  |  | 596.6 | 90.2 |
|  |  |  | A1 |  |  | 812.4 | 90.0 |
|  |  |  | A2 |  |  | 1466.3 | 82.0 |
|  |  |  | A3 |  |  | 786.4 | 92.9 |
|  |  |  | F1 |  |  | 2602 | 77.1 |
|  |  |  | F2 |  |  | 2519.9 | 78.1 |
|  |  |  | F3 |  |  | 1915.6 | 96.9 |
|  |  |  | F4 |  |  | 2053.3 | 96.4 |
|  |  |  | F5 |  |  | 967.4 | 90.7 |
|  |  |  | F6 |  |  | 2130 | 91.4 |
|  | *Pokrovsky et al.*, (2005) | River Bed Sediments | 53 RBS | 248 | -9 | 475 | 58.2 |
|  |  |  | 54 RBS |  |  |  | 48.8 |
|  |  |  | 55 RBS |  |  |  | 47.7 |

**Table s3**. Terrestrial geochemistry

|  |  |  | SV10A mudstone | LG001 mudstone | OS001 mudstone | Idaho NP004 |
| --- | --- | --- | --- | --- | --- | --- |
| 0% clay | siltstone | 0 | 47.41 | 44.69 | 47.51 | 58.62 |
| 1% clay |  | 1 | 47.52 | 44.90 | 47.67 | 58.80 |
| 33% clay | mudstone | 33 | 50.80 | 51.43 | 51.67 | 64.08 |
| 66% Clay | claystone | 66 | 53.77 | 57.90 | 54.35 | 68.93 |
| 100% Clay |  | 100 | 56.48 | 64.29 | 66.65 | 73.41 |
|  |  |  |  |  |  |  |

**Table s4**. Mudstone models

|  |  | Deopositional Site | CIA |
| --- | --- | --- | --- |
| Haridwar | G1B | Bed Load Sediments | 49.2 |
| Nagal | G2B | Bed Load Sediments | 51.3 |
| Bijnor | G3B | Bed Load Sediments | 52.5 |
| Doranagar | G4B | Bed Load Sediments | 52.3 |
| Tigri | G5B | Bed Load Sediments | 55.6 |
| Soron | G8B | Bed Load Sediments | 52.0 |
| Fatchgarh | G10B | Bed Load Sediments | 50.5 |
| Kannauj | G11B | Bed Load Sediments | 56.7 |
| Bilhaur | G12B | Bed Load Sediments | 55.3 |
| Kanpur | G13B | Bed Load Sediments | 55.1 |
| Manda | G14B | Bed Load Sediments | 53.0 |
| Mizapur | G15B | Bed Load Sediments | 55.8 |
| Haridwar | G1AFP | Active Flood Plain | 62.6 |
| Bijnor | G3AFP1 | Active Flood Plain | 55.4 |
| Bijnor | G3AFP2 | Active Flood Plain | 55.0 |
| Doranagar | G4AFP | Active Flood Plain | 50.5 |
| Tigri | G5AFP | Active Flood Plain | 58.6 |
| Anupsahar | G6AFP1 | Active Flood Plain | 56.3 |
| Anupsahar | G6AFP2 | Active Flood Plain | 54.2 |
| Naurora | G7AFP | Active Flood Plain | 56.9 |
| Soron | G8AFP | Active Flood Plain | 54.2 |
| Kampil | G9AFP | Active Flood Plain | 54.4 |
| Fatchgarh | G10AFP | Active Flood Plain | 54.7 |
| Bilhaur | G12AFP | Active Flood Plain | 63.8 |
| Kanpur | G13AFP | Active Flood Plain | 57.3 |
| Manda | G14AFP1 | Active Flood Plain | 66.0 |
| Manda | G14AFP2 | Active Flood Plain | 61.8 |
| Nagal | G2IF | Interfluve (Older Flood Plain) | 68.2 |
| Bijnor | G3IF | Interfluve (Older Flood Plain) | 74.0 |
| Tigri | G5IF | Interfluve (Older Flood Plain) | 53.6 |
| Fatchgarh | G10IF | Interfluve (Older Flood Plain) | 60.4 |
| Kannauj | G11IF | Interfluve (Older Flood Plain) | 53.9 |
| Kanpur | G13IF | Interfluve (Older Flood Plain) | 56.3 |
| Manda | G14IF | Interfluve (Older Flood Plain) | 59.2 |
| Mizapur | G15IF1 | Interfluve (Older Flood Plain) | 65.0 |
| Mizapur | G15IF2 | Interfluve (Older Flood Plain) | 53.6 |

**Table s5**. Depositional Site CIA values

Reference:

*Singh* (2009)

**Supplementary References**

Singh, P., 2009. Major, trace and REE geochemistry of the Ganga River sediments: influence of provenance and sedimentary processes. *Chemical geology*, *266*(3-4), pp.242-255. doi: 10.1016/j.chemgeo.2009.06.013
